# CypA Mediates Non‐Small Cell Lung Cancer Chemoresistance by Attenuating Ferroptosis via Stabilizing SLC7A11

**DOI:** 10.1002/advs.202511947

**Published:** 2025-11-07

**Authors:** Zhongcheng Wang, An Li, Ziwei Song, Xiangming Liu, Yong Ge, Zhiqiao Chen, Yuhui Liu, Boyu Zhang, Hao Zhang, Ting Lan

**Affiliations:** ^1^ Xuzhou Key Laboratory of Laboratory Diagnostics Xuzhou Medical University Xuzhou Jiangsu 221004 China; ^2^ Department of Pathophysiology School of Basic Medical Sciences Xuzhou Medical University Xuzhou Jiangsu 221004 China; ^3^ School of Medical Technology Xuzhou Medical University Xuzhou Jiangsu 221004 China; ^4^ Department of Laboratory Medicine The Second Affiliated Hospital of Nanjing University of Chinese Medicine 23 Nanhu Road Nanjing Jiangsu 210017 China; ^5^ Department of Thoracic Surgery Affiliated Hospital of Xuzhou Medical University 99 West Huaihai Road Xuzhou Jiangsu 221006 China

**Keywords:** CypA, chemoresistance, ferroptosis, non‐small cell lung cancer, SLC7A11

## Abstract

Non‐small cell lung cancer (NSCLC) remains a major oncological challenge due to intrinsic or acquired chemoresistance, underscoring the urgent need to decipher novel regulatory mechanisms. Here, cyclophilin A (CypA) is identified as a critical mediator of cisplatin (DDP)/paclitaxel (DTX) resistance in NSCLC by suppressing ferroptosis, an iron‐dependent form of regulated cell death. CypA is significantly overexpressed in DDP/DTX‐resistant NSCLC cell lines and patient tissues, correlating with poor prognosis. Mechanistically, CypA stabilizes the ferroptosis suppressor SLC7A11 by competitively binding to its K37 site, blocking ubiquitination and proteasomal degradation mediated by the E3 ligase TRIM3. This interaction maintains cystine uptake, glutathione biosynthesis, and redox homeostasis, thereby attenuating lipid peroxidation and ferroptosis induction by chemotherapeutics. knockout of CypA or pharmacological inhibition with cyclosporine A (CsA) reverse resistant NSCLC cells to DDP/DTX both in vitro and in vivo by restoring ferroptosis. Combined CsA and chemotherapy treatment significantly enhances tumor regression, as evidenced by increased 4‐HNE and reduced SLC7A11 expression in vivo. The study uncovers a CypA/SLC7A11/TRIM3 axis governing ferroptosis evasion in NSCLC chemoresistance and highlights CypA as a promising therapeutic target. Repurposing CsA to inhibit CypA represents a translatable strategy to overcome chemotherapy resistance, offering preclinical validation for improving outcomes in NSCLC patients.

## Graphical abstract

Summary diagram for the role of CypA in chemoresistance in non‐small cell lung cancer (NSCLC). CypA is overexpressed in chemoresistance NSCLC and drives therapeutic evasion by competitively binding to the K37 site of SLC7A11, thereby blocking TRIM3 mediated K11‐linked ubiquitination and proteasomal degradation, ultimately inhibiting lipid peroxidation and ferroptosis. Administration in combination with CypA inhibitor can effectively restore the sensitivity of NSCLC patients to chemoresistance.

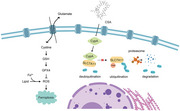

## 1 Introduction

Lung cancer is one of the most common and fatal cancers and ≈2.48 million new lung cancer cases accounting for 12.4% in worldwide.^[^ ^1^ ^]^ Non‐small cell lung cancer (NSCLC) accounts for the vast majority (≈85%) of lung cancers, and the predicted 5‐year survival rate is only 15.9%.^[^ ^2^ ^]^ Despite the huge advances in treatment options including surgery, chemotherapy, radiation and targeted therapies, prognosis remains poor because of the presence of locally advanced metastatic tumors in most patients at the time of diagnosis.^[^ ^3^ ^]^ NSCLC patients undergoing surgery and treated uniformly with a one‐size‐fits‐all approach‐Platinum‐based chemotherapy, where cisplatin or carboplatin is used in combination with gemcitabine, vinorelbine, pemetrexed or taxanes (docetaxel or paclitaxel) to eradicate this micrometastatic disease, but this therapy leads to ≈5% survival benefit only.^[^ ^4^ ^]^ Newer targeted agents are benefitting a small fraction of patients with NSCLC with lung adenocarcinoma (LUAC) subtypes, while personalized treatment options for lung squamous cell carcinoma (LUSC) subtypes are absent.^[^ ^5^ ^]^ In addition, although the field of NSCLC has progressed remarkably including immune checkpoint blockers (ICBs) and personalized targeted therapies for oncogene‐addicted NSCLC (the presence of a single driver mutation necessary for tumor survival), these newer targeted and immune therapies lack sustained effects due to inherent or acquired drug resistance.^[^ ^6^ ^]^ Therefore, identifying targets to overcome drug resistance holds great promise for preventing lethal recurrences in patients with NSCLC in the future.^[^ ^7^, ^8^ ^]^

Ferroptosis, an iron‐dependent, lipid peroxidation‐driven form of regulated cell death, has emerged as a pivotal player in cancer biology, particularly in the context of tumor resistance to therapy.^[^ ^9^ ^]^ The evasion of ferroptosis in drug‐resistant tumors often involves upregulation of antioxidant defenses, most notably the SLC7A11/glutathione (GSH)/glutathione peroxidase 4 (GPX4) axis. As a core regulator of ferroptosis, SLC7A11 is capable of exporting intracellular glutamate and importing extracellular cystine, which neutralizes reactive oxygen species (ROS) and supports GPX4‐mediated detoxification of lipid peroxides, thereby inhibiting ferroptosis.^[^ ^10^ ^]^ The expression of SLC7A11 is upregulated in multiple cancers.^[^ ^11^, ^12^, ^13^, ^14^ ^]^ Transcription of SLC7A11 could be induced by transcription factors. In lung cancer, the RNA‐binding protein RBMS1 directly interacts with the translation initiation factor eIF3d, thereby bridging the 3′‐untranslated region (3′UTR) and 5′‐untranslated region (5′UTR) of SLC7A11 mRNA to enhance its expression.^[^ ^15^ ^]^ Concurrently, transcription factors ATF4 and SOX2 promote SLC7A11 expression by regulating its promoter region.^[^ ^16^ ^]^ Wild type p53 suppresses SLC7A11 transcription, reduces cystine uptake, and promotes lipid peroxidation and ferroptosis.^[^ ^17^ ^]^ Apart from transcriptional regulation, SLC7A11 expression is also controlled at the post‐translational level. In endometrial cancer, NSUN2 epigenetically regulates SLC7A11 mRNA through mC modification, enhancing its stability and elevating its expression levels.^[^ ^18^ ^]^ SOCS2 acts as an adaptor to transfer ubiquitin to SLC7A11, promoting K48‐linked polyubiquitination and degradation of SLC7A11, ultimately triggering ferroptosis and enhancing radiosensitivity in hepatocellular carcinoma.^[^ ^19^ ^]^ Conversely, EFNA4 recruits the deubiquitinase USP9X to inhibit SLC7A11 degradation, thereby suppressing ferroptosis and augmenting the proliferation and metastatic capacity of hepatocellular carcinoma.^[^ ^20^ ^]^ TRIM3, functioning as an E3 ubiquitin ligase for SLC7A11, binds to and promotes its ubiquitin‐dependent degradation in non‐small cell lung cancer.^[^ ^21^ ^]^ In ovarian cancer, however, the deubiquitinases DUBA and DTUD5 stabilize SLC7A11 expression by interacting with it.^[^ ^22^ ^]^

The cyclophilin family, highly conserved in humans, participates in protein folding and trafficking through its peptidylprolyl isomerase (PPIase) domain.^[^ ^23^ ^]^ Studies have shown that cyclophilin A (CypA) is significantly overexpressed in gastric, hepatocellular, NSCLC, colorectal, and esophageal squamous cell carcinomas, where it regulates tumor proliferation, invasion, stemness, and epithelial‐mesenchymal transition (EMT).^[^ ^24^ ^]^ In colorectal cancer, CypA knockdown suppresses tumor cell migration by inhibiting EMT and the p38 pathway.^[^ ^25^ ^]^ CypA also protects A549 cells from H_2_O_2_‐induced oxidative stress and apoptosis via activation of the PI3K/Akt/mTOR pathway.^[^ ^26^ ^]^ Notably, CypA engages in nuclear signaling by binding β‐catenin and promoting its nuclear translocation, thereby amplifying TCF4‐mediated transcriptional activation of Wnt/β‐catenin signaling to enhance glioma stemness and radioresistance.^[^ ^27^ ^]^ Intriguingly, under oxidative stress, CypA exerts redox enables peroxiredoxin‐2 (PRDX2) mediated cysteine redox cycling to reduce intracellular reactive oxygen species (ROS) levels, elicits chemoresistance in colorectal cancer.^[^ ^28^ ^]^ In NSCLC, CypA competitively interacts with Kelch‐like ECH‐associated protein 1 (KEAP1), disrupting its ability to promote nuclear factor erythroid 2‐related factor 2 (Nrf2) ubiquitination, thus modulating glutamine metabolism.^[^ ^29^ ^]^ Our previous studies in ovarian cancer revealed elevated CypA expression and identified a novel TAF15/STAT5A/miR‐514a‐3p regulatory feedback loop. This pathway drives EMT progression, promotes tumor growth and metastasis, and facilitates extracellular CypA release.^[^ ^30^ ^]^ However, the role of CypA in chemotherapy responses of lung cancer remains unknown.

Here, we aim to identified the role of CypA in NSCLC chemoresistance. CypA is overexpressed in cisplatin (DDP) and paclitaxel (DTX) resistant NSCLC and the high expression of CypA is correlated with poor patient prognosis. Knockout of CypA impeded tumor cell survival by promoting cellular ferroptosis. CypA competitively binds to the K37 site of SLC7A11 with TRIM3, thereby inhibiting TRIM3‐induced ubiquitination of SLC7A11 and stabilizing its expression. Furthermore, the inhibitor of CypA, cyclosporine A (CsA), targeting CypA to reverse resistance. Our study seeks to uncover novel mechanistic insights into ferroptosis regulation in NSCLC and provide a preclinical rationale for overcoming chemotherapy resistance through CypA inhibition.

## 2 Results

### 2.1 CypA is Positively Correlated with Non‐Small Cell Lung Cancer Progression

**Figure 1:**
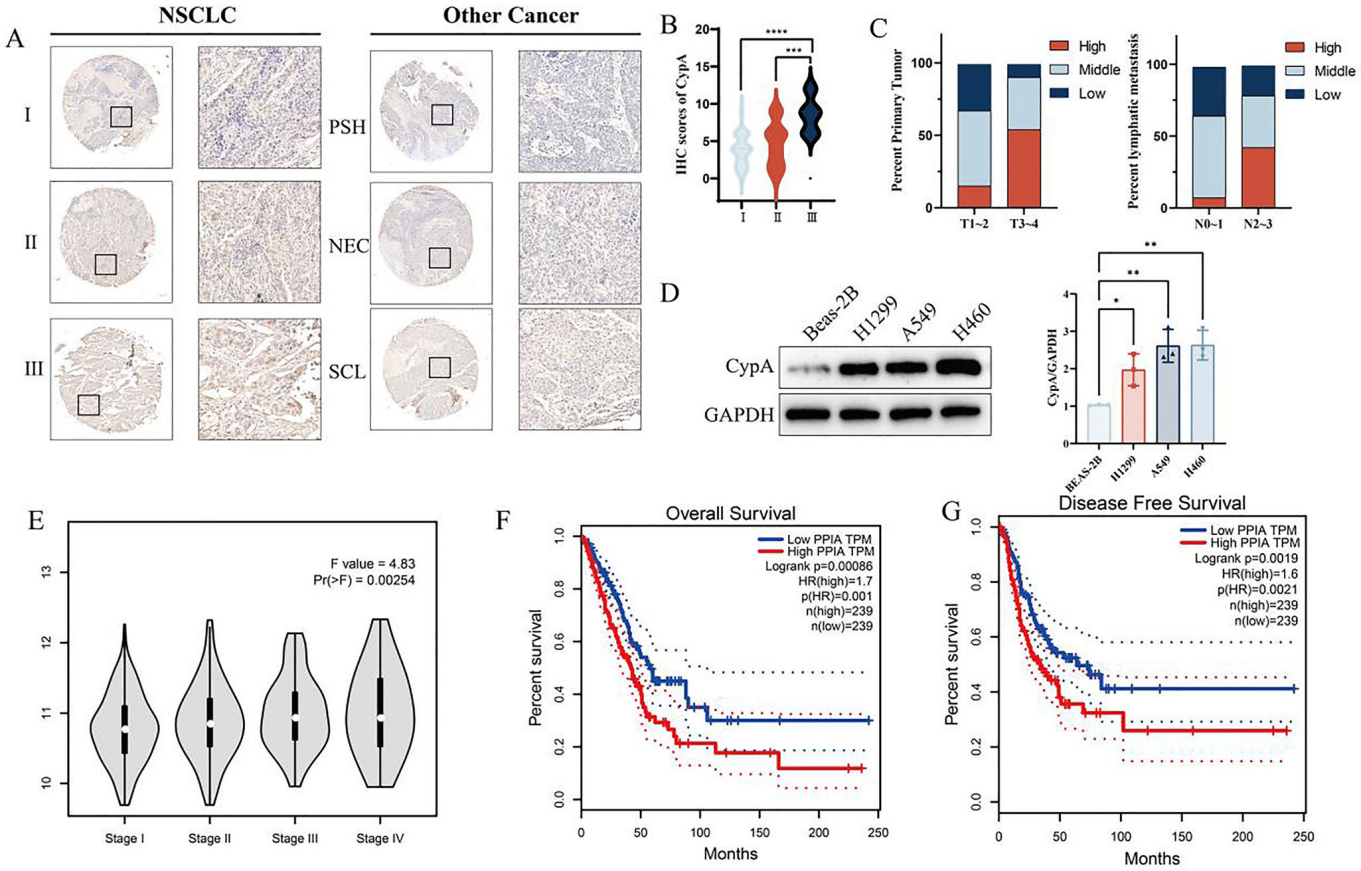
CypA is positively correlated with non‐small cell lung cancer progression. A,B) IHC analysis of CypA in different stages of NSCLC tissue and PSH, NEC and SCL tissue. Scale bar: 300 µm (left), 30 µm (right). (A) is the representative images and (B) is the quantifications. PSH: pulmonary sclerosing hemangioma, NEC: neuroendocrine carcinoma, and SCL: small cell lung carcinoma. C) Correlation between CypA expression and the percentage distribution of primary tumor and lymphatic metastasis across tumor‐node staging categories. D) Western blot analysis the expression of CypA in NSCLC cells (H1299, A549 and H460) and normal ovarian cell (BEAS‐2B). E the mRNA levels of CypA in different stages of NSCLC. F) Kaplan‐Meier analysis of the overall survival of OC patients associated with CypA expression from the GEPIA database. G) Kaplan‐Meier analysis of the progression‐free survival of OC patients associated with CypA expression. Data are represented as the mean ± SD. Statistical analysis was performed using Student’ s *t*‐test, *p < 0.05; ***p < 0.001; ****p < 0.0001.

To determine the expression of CypA in lung cancer, we analyzed the tissues of 62 lung cancer patients by immunohistochemical microarrays. In NSCLC, CypA expression levels are significantly elevated compared to those observed in pulmonary sclerosing hemangioma (PSH), neuroendocrine carcinoma (NEC), and small cell lung carcinoma (SCLC) (**Figure** **1**A). Furthermore, this increased CypA expression demonstrates a positive correlation with NSCLC progression (Figure 1E). Analysis of the TCGA database comprising 477 patients with non‐small cell lung cancer (NSCLC) revealed that CypA expression was positively correlated with the Tumor, Node, and Metastasis (TNM) stage (**Table** **1**). Furthermore, a correlation analysis of tumor characteristics and Cyclophilin A (CypA) expression in 60 NSCLC patients demonstrated that elevated CypA levels were more prevalent in cases with larger tumor dimensions and higher lymph node metastasis scores (Figure 1B,C). Next, we also determined CypA expression in a panel of NSCLC cell lines. As expected, the protein levels in normal cell line, BEAS‐2B cells (human lung epithelial cell), were markedly lower than those in tumor cells (Figure 1D). Moreover, NSCLC patients with high CypA expression displayed a shorter overall survival and progression‐free survival than those with low CypA expression (Figure 1F,G). And multivariate Cox regression analysis revealed that CypA are independent prognostic factors (Figure S1H, Supporting Information). Taken together, these data indicated higher CypA levels are linked to increased aggressiveness of NSCLC.

**Table 1:** Correlation between PPIA and NSCLC variable.

Variable		N=477	Expression	P value
T				0.010063
	T1	63(13.21%)	5.467957	
	T1a	46(9.64%)	5.420018	
	T1b	53(11.11%)	5.351127	
	T2	145(30.40%)	5.545082	
	T2a	80(16.77%)	5.534953	
	T2b	27(5.66%)	5.415626	
	T3	43(9.01%)	5.496203	
	T4	17(3.56%)	5.688976	
	TX	3(0.63%)	5.403193	
N				2.53E‐06
	N0	307(64.36%)	5.441035	
	N1	90(18.87%)	5.601681	
	N2	67(14.05%)	5.606329	
	N3	2(0.42%)	6.056069	
	NX	11(2.31%)	5.221863	
M				0.000947
	M0	311(65%)	5.520661	
	M1	166(35%)	5.743166	
Gender				0.744933
	MALE	256(53.7%)	5.498034	
	FEMALE	221(46.3%)	5.486954	
Age				0.001581
	Young(<60)	132(27.7%)	5.578385	
	Old(>60)	345(72.3%)	5.459069	
Status				2.09E‐05
	Alive	302(63%)	5.437598	
	Dead	175(37%)	5.586121	

### 2.2 CypA Upregulates the Expression of SLC7A11 and Confers Resistance Against Ferroptosis

**Figure 2:**
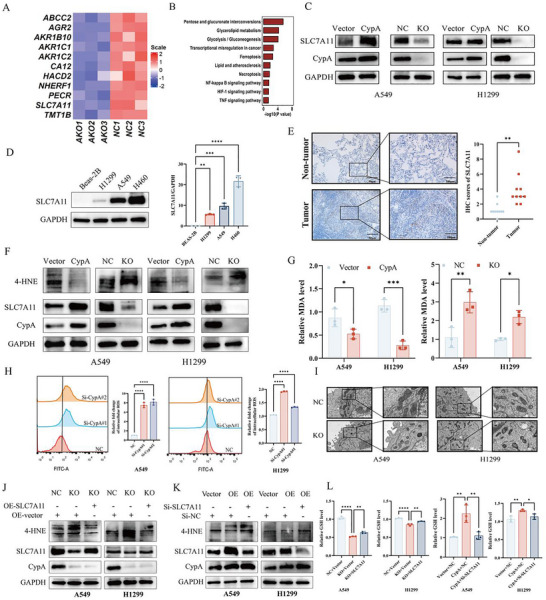
CypA upregulates the expression of SLC7A11 and confers resistance against ferroptosis. A) Mass spectrometry analysis identified the proteins that CypA knockdown. B) KEGG pathway analysis of signaling pathways affected by CYPA knockout. C) Western blot analysis SLC7A11 expression after CypA overexpression or knockout in A549 and H1299. D) Western blot analysis the expression of SLC7A11 in NSCLC cells (H1299, A549 and H460) and normal ovarian cell (BEAS‐2B). E) IHC analysis of SLC7A11 in lung cancer and non‐tumorous lung tissue. Scale bar: 300 µm (left), 30 µm (right). F) Expression of MDA after CypA overexpression or knockout in A549 and H1299. G) Expression of GSH after CypA overexpression or knockout in A549 and H1299. H) Flow cytometry analysis of ROS levels after CypA overexpression or knockout in A549 and H1299. I) Transmission electron microscopy analysis of mitochondrial structure after CypA knockout in A549 and H1299 cells. J) Expression of 4‐HNE transfected with NC, CypA knockout or SLC7A11 overexpression plus CypA knockout. K) Expression of 4‐HNE transfected with NC, CypA overexpression or SLC7A11 knockdown plus CypA overexpression. Data are represented as the mean ± SD (*n* = 3). Statistical analysis was performed using Student’ s *t*‐test, *p < 0.05; **p < 0.01; ***p < 0.001; ****p < 0.0001.

To determine the mechanism of CypA in NSCLC progression, LC‐MS was performed to analyze proteomic differences between the control group and the CypA knocked out NSCLC cell line by CRISPR/Cas9 gene editing. Proteomic data showed that, with the knocked out CypA, SLC7A11 was significantly downregulated in NSCLC cell line (**Figure** **2**A) and the ENCORI database also found that CypA was positively correlated with SLC7A11 expression in NSCLC (Figure S1A, Supporting Information). KEGG analysis revealed that the ferroptosis signaling pathway was the critical pathway activated by the knocked out of CypA (Figure 2B). Western blot analysis confirmed that the expression of SLC7A11 was increased in the H1299 and A549 cell lines with overexpression of CypA, while it was decreased with the knockout of CypA (Figure 2C). Furthermore, the CypA inhibitor CSA suppressed SLC7A11 expression in NSCLC cells in a concentration‐dependent manner (Figure S1B, Supporting Information). Additionally, we confirmed that SLC7A11 is overexpressed in NSCLC cell lines (H1299, A549, H460) compared with normal Lung epithelial cells (Beas‐2B) (Figure 2D). And analysis of the TCGA database comprising 477 patients with NSCLC revealed that SLC7A11 expression was positively correlated with the Node, and Metastasis stage (**Table** **2**). SLC7A11 was also overexpressed in NSCLC patients compared with normal lung tissues (Figure 2E).

**Table 2:** Correlation between SLC7A11 and NSCLC variable.

Variable		N=477	Expression	P value
T				0.141557
	T1	63(13.21%)	1.838942	
	T1a	46(9.64%)	1.871545	
	T1b	53(11.11%)	2.139946	
	T2	145(30.40%)	2.186021	
	T2a	80(16.77%)	1.841046	
	T2b	27(5.66%)	2.152398	
	T3	43(9.01%)	2.340758	
	T4	17(3.56%)	2.353232	
	TX	3(0.63%)	2.032485	
N				0.19768
	N0	307(64.36%)	2.008051	
	N1	90(18.87%)	2.185352	
	N2	67(14.05%)	2.237914	
	N3	2(0.42%)	0.864492	
	NX	11(2.31%)	1.787753	
M				0.089734
	M0	311(65%)	2.054152	
	M1	166(35%)	2.6587	
Gender				0.000269
	MALE	256(53.7%)	2.270163	
	FEMALE	221(46.3%)	1.885867	
Age				0.549985
	Young(<60)	132(27.7%)	2.012738	
	Old(>60)	345(72.3%)	2.083497	
Status				0.001752
	Alive	302(63%)	1.938388	
	Dead	175(37%)	2.280542	

As a core regulator of ferroptosis, SLC7A11 inhibits ferroptosis by exporting intracellular glutamate and importing extracellular cystine to sustain redox homeostasis.^[^ ^31^ ^]^ This process supports GPX4 redox activity, reduces intracellular lipid peroxides or organic hydroperoxides to alcohols, and diminishes ROS accumulation, ultimately mitigating ferroptosis.^[^ ^11^ ^]^ Although our prior findings indicate that CypA deficiency reduces SLC7A11 expression, it does not significantly affect GPX4 expression, implying that CypA enhances SLC7A11 expression, thereby increasing GPX4 activity and suppressing ferroptosis (Figure S1C, Supporting Information). As expected, forced expression of CypA led to A549 and H2122 cells resistant to RSL‐induced cell death, while knockout of CypA is opposite (Figure S1D, Supporting Information). Moreover, knockout of CypA led to elevated lipid peroxidation, ROS levels and free iron levels in A549 and H2122 cells. In contrast, overexpression CypA pare lipid peroxidation and free iron levels (Figure 2F–H; Figure S1E‐G, Supporting Information). Furthermore, transmission electron microscopy (TEM) showed a shrunken morphology of mitochondria in knockout of CypA cells (Figure 2I). To determine the role of the CypA/xCT axis in the ferroptosis of NSCLC, we performed rescue experiments and found that overexpression of SLC7A11 rescued CypA knockout‐induced ferroptosis, whereas knockdown of SLC7A11 in overexpression CypA cell lines conversely exacerbated ferroptosis (Figure 2J–L).

### 2.3 CypA Interacts with SLC7A11 and Stabilizes its Expression via the Inhibition of its Ubiquitination

**Figure 3:**
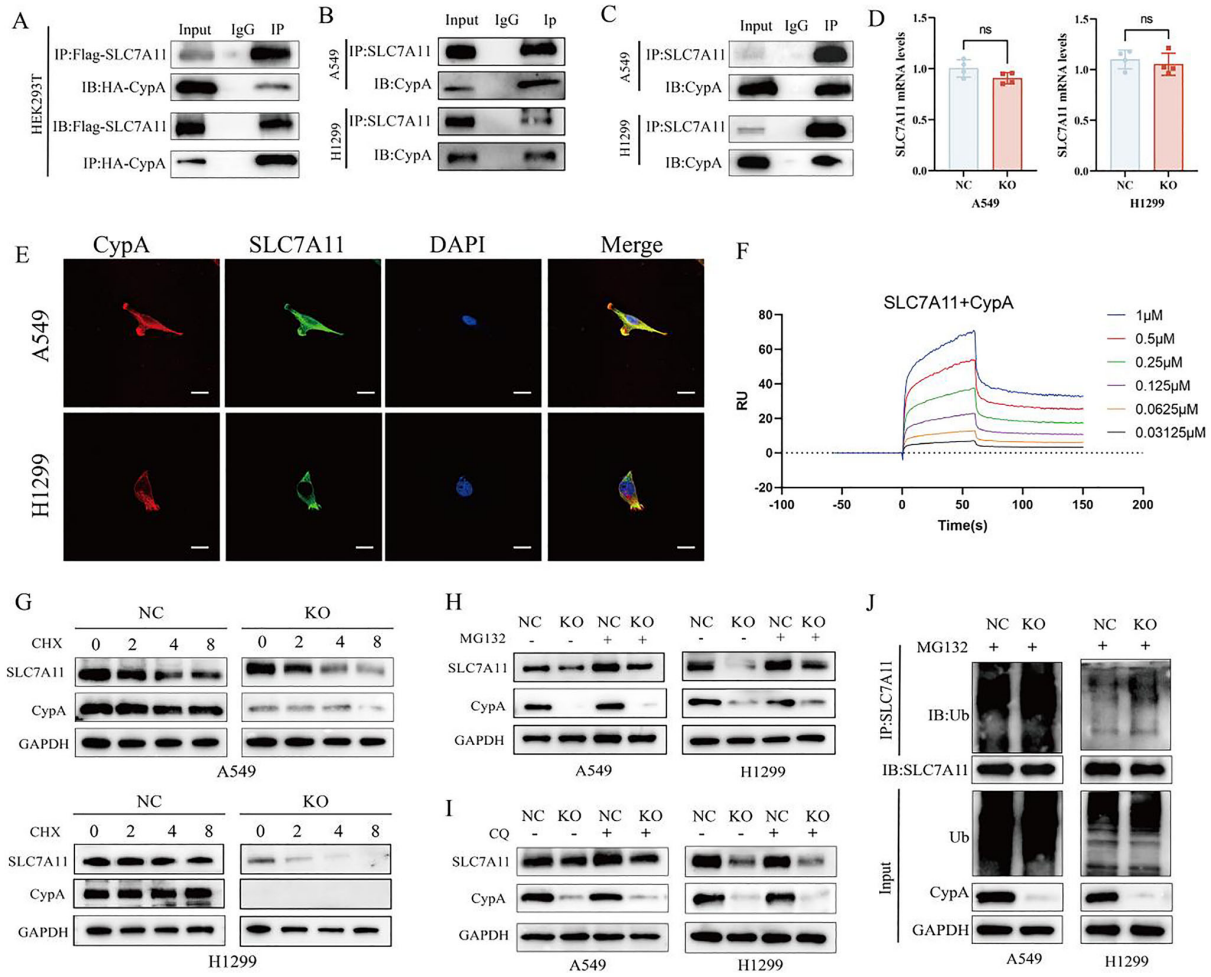
CypA interacts with SLC7A11 and stabilizes its expression via the inhibition of its ubiquitination. A) HEK293 cells transfected with HA‐CypA and Flag‐xCT were subjected to IP analysis with anti‐Flag antibody and then analyzed by IB with the indicated antibodies. B,C) The interaction of CypA and SLC7A11 in A549 and H1299 by Co‐IP. D) qRT‐PCR analysis of SLC7A11 mRNA levels after CypA knockout. E) Confocal assay showing co‐localization of CypA (red) and xCT (green) in A549 and H1299 cells. Nuclei were counterstained with DAPI (blue). Scale bar: 10 µm. F) The binding between SLC7A11 and varying concentrations of CypA was assessed by SPR. G) Western blot analysis xCT protein levels in A549 and H1299 cells treated with CHX after CypA knockout. H) Western blot analysis xCT protein levels in A549 and H1299 cells treated with MG132 after CypA knockout. I) Western blot analysis xCT protein levels in A549 and H1299 cells treated with CQ after CypA knockout. J) IP and Western blot analysis the ubiquitination level of xCT in A549 and H1299 cells treated with MG132 (20 µM) for 6 h before collection after CypA knockout.

To further investigate the underlying mechanisms by CypA regulates SLC7A11 expression, we first investigated the interaction between CypA and SLC7A11 using co‐immunoprecipitation. HEK293T cells were co‐transfected with Flag‐tagged SLC7A11 and HA‐tagged CypA. The anti‐Flag immunoprecipitates were then probed with an anti‐Flag antibody. Exogenous Flag‐SLC7A11 was detected in the anti‐HA immunoprecipitates (**Figure** **3**A). Subsequently, we confirmed in H1299 and A549 cells that endogenous CypA could co‐immunoprecipitate with endogenous SLC7A11 (Figure 3B,C). Confocal analysis determined the co‐localization of these proteins (Figure 3E). However, qRT‐PCR analysis demonstrated that there was no change of SLC7A11 mRNA in knockout or overexpression of CypA compared with control cells (Figure 3D). Surface plasmon resonance (SPR) experiments further demonstrated that CypA exhibits a strong, dose‐dependent affinity for SLC7A11 (dissociation constant, KD = 4.22E‐07) (Figure 3F). Moreover, we performed a half‐life assay by treating CypA knockout cells with cycloheximide (CHX). The results showed that CypA enhanced the stability of SLC7A11 (Figure 3G), suggesting a post‐translational regulation of CypA on SLC7A11. To determine the degradation pathway of SLC7A11 regulated by CypA, we treated A549 and H1299 cells stably expressing control or CypA knockout with DMSO, lysosome inhibitor Chloroquine (CQ), or proteasome inhibitor MG132. Results showed that the expression of SLC7A11 was only restored by MG132 under CypA deficiency (Figure 3H,I). For a more intuitive study of the effect of CypA on SLC7A11 ubiquitination, endogenous CO‐IP was performed to test SLC7A11 ubiquitination level. In NSCLC cells, CypA decreased SLC7A11 overall polyubiquitination (Figure 3J). These results suggested that CypA stabilizes SLC7A11 protein expression by inhibiting the SLC7A11 proteasomal degradation pathway.

### 2.4 CypA and TRIM3 Competitively Bind to the K37 Site of SLC7A11

**Figure 4:**
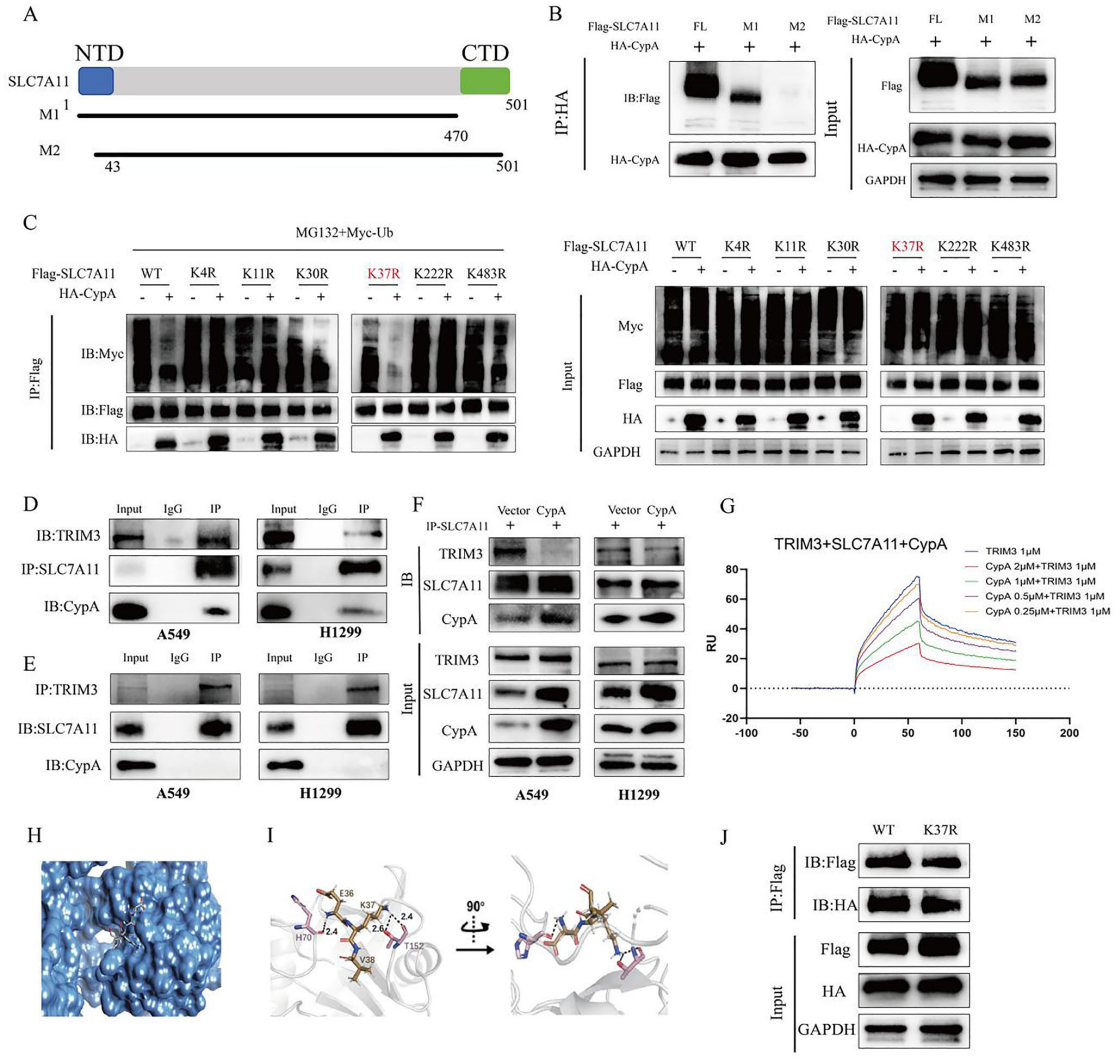
CypA and TRIM3 competitively bind to the K37 site of SLC7A11. A) Sketch map of full‐length (FL) Flaglabeled xCT, and its deletion mutants. B) HEK293 cells were co‐transfected with HA‐CypA and FL Flag‐xCT or deletion mutants. After treatment with MG132 (20 µM) for 6 h, cell lysates were subjected to IP followed by western blot assay with the indicated antibodies. C) HEK293 cells transfected with HA‐CypA together with ubiquitin WT or different Flag‐xCT mutants were subjected to IP with anti‐Flag antibody and analyzed by western blotting. Cells were treated with MG132 (20 µM) for 6 h before collection. D) CO‐IP analysis detects the CypA and TRIM3 were subjected to IP with anti‐xCT antibody in A549 and H1299 cells. E) CO‐IP analysis detects the xCT and TRIM3 were subjected to IP with anti‐CypA antibody in A549 and H1299 cells. F) CO‐IP analysis detects the CypA and TRIM3 were subjected to IP with anti‐xCT antibody after CypA overexpression in A549 and H1299 cells. G) SLC7A11was coupled on the chips. The binding between TRIM3 and SLC7A11 in the varying concentrations of CypA was assessed by SPR. H) Crystal structure of CypA in complex with the SLC7A11 fragment(36EKV38) CypA Binding Motif. I) CypA is shown in gray cartoon, and the interacted residues are displayed as salmon sticks. The SLC7A11 fragment is presented as slate sticks. The black dashed line denotes the hydrogen contact. J) CO‐IP analysis the interaction between xCT (Flag) and CypA (HA) using an anti‐Flag antibody in HEK293 cells transfected with either xCT WT or xCT K37R.

To determine the specific domains of SLC7A11 that mediate its interaction with CypA, we generated Flag‐tagged truncated forms of SLC7A11 (**Figure** **4**A). HEK293T cells were co‐transfected with HA‐CypA and the Flag‐tagged truncations of SLC7A11. Anti‐HA immunoprecipitates were probed using the anti‐Flag antibody. The results revealed that the N‐terminal sequences of xCT mediated the interaction between SLC7A11 and CypA (Figure 4B).

Furthermore, we predicted six putative ubiquitination sites of xCT using the PhosphosSitePlus website (Figure S2A, Supporting Information).^[^ ^31^ ^]^ To further confirm which lysine residue of SLC7A11 was deubiquitinated, we mutated the potential lysine residues on SLC7A11. Our data demonstrated that co‐expression of a panel of xCT mutants with ubiquitin revealed minimal ubiquitination of the K37R mutant at the protein level, as determined by in vitro ubiquitination assays (Figure 4C; Figure S2B, Supporting Information). Consistent with prior studies, this work further demonstrates that TRIM3 acts as the specific E3 ubiquitin ligase for SLC7A11 in NSCLC, mediating K37 site‐dependent ubiquitination to drive its proteasomal degradation. CypA, as a peptidylprolyl cis‐trans isomerase activity protein, how to regulate SLC7A11 ubiquitination? We then asked whether CypA affected the interaction of TRIM3 and SLC7A11. Co‐immunoprecipitation analysis revealed that SLC7A11 binds both TRIM3 and CypA, but TRIM3 does not directly bind CypA (Figure 4D,E). Moreover, overexpression of CypA profoundly impaired the binding of TRIM3 and SLC7A11 (Figure 4F). This result confirmed that CypA suppressed the binding of TRIM3 and SLC7A11. And the SPR also revealed that CypA was able to prevent SLC7A11 and TRIM3 binding (Figure 4G). Molecular docking and molecular dynamics simulation analysis revealed that CypA binds to the K37 site of SLC7A11 (Figure 4H,I). Consistently, mutation of the K37 in SLC7A11 significantly weakened its binding to CypA compared with the wild type (Figure 4J). Taken together, these results indicate that CypA protects SLC7A11 from TRIM3‐mediated ubiquitination and degradation by binding to its K37 site, thereby stabilizing SLC7A11 expression.

### 2.5 High Expression of CypA and SLC7A11 Predicts Poor Response to Chemotherapy in NSCLC

**Figure 5:**
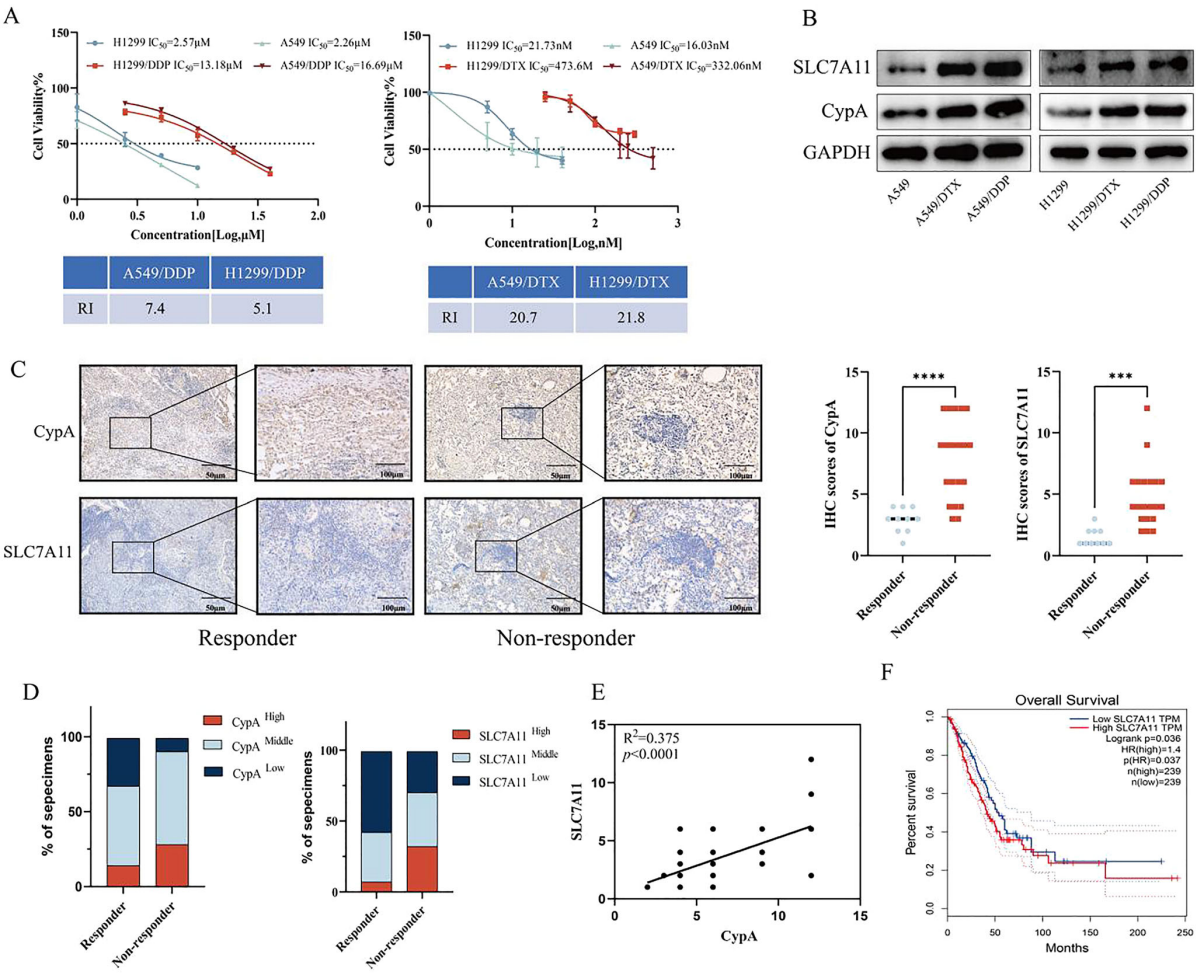
High expression of CypA and SLC7A11 predicts poor response to chemotherapy in NSCLC. A) Dose‐response curves of parental NSCLC cell lines and corresponding resistant NSCLC cell lines treated with indicated concentrations of cisplatin (DDP) or docetaxel (DTX) for 48 h. B) Western blot analysis of CypA and SLC7A11expression in the parental and resistant A549 and H1299 cell lines. C) IHC images for CypA and SLC7A11 expression in NSCLC tumor tissue samples with different responses to chemotherapy. Scale bars: 100 mm (left) and 20 mm (right). D) Analysis of the percentage of specimens with low, middle or high CypA and SLC7A11 expression, relative to the response to chemotherapy by Pearson chi‐square test. E) The correlation between CypA and SLC7A11 was analyzed by Pearson correlation test. F) Overall survival of patients with NSCLC with low or high CypA expression according to the cohort GEPIA (p = 0.037, log‐rank test). Data are represented as the mean ± SD (*n* = 3). Statistical analysis was performed using Student’ s *t*‐test, ***p < 0.001; ****p < 0.0001. See also Table S1 (Supporting Information).

Given the role of CypA in stabilizing SLC7A11 expression to resist ferroptosis, we wondered whether CypA is implicated in NSCLC resistance to chemotherapy. As shown in **Figure** **5**A,B, the protein levels of CypA and SLC7A11 were upregulated in chemoresistant NSCLC cell lines (A549/DDP, A549/DTX, H1299/DDP, H1299/DTX) compared to that in parental cells (A549, H1299). The confocal results further confirmed that the expression and co‐localization of CypA and SLC7A11 were increased in chemoresistant NSCLC cell lines, while the co‐localization of SLC7A11 and TRIM3 was decreased (Figure S2C, Supporting Information). To further clarify the clinical relevance of the CypA‐SLC7A11 axis in NSCLC chemoresistance, a cohort using specimens from 30 cases of NSCLC (see details in **Table** **3**) was categorized into the responder or non‐responder group based on the sensitivity to chemotherapy. Immunohistochemistry (IHC) analysis revealed overall increased protein levels of CypA and SLC7A11 in NSCLC tissues from the non‐responder group (Figure 5C,D). Intriguingly, the protein levels of CypA and SLC7A11 were positively correlated in the NSCLC tissues (Figure 5E). And multivariate Cox regression analysis revealed that SLC7A11 are independent prognostic factors (Figure S1H, Supporting Information). Moreover, the protein levels of SLC7A11 were positively correlated with the overall survival rate in patients with NSCLC from the GEPIA (Figure 5F; Figure S2D‐E, Supporting Information), indicating the prognostic value of SLC7A11 in NSCLC. Taken together, these results suggested that CypA and SLC7A11 predict poor clinical response to chemotherapy as well as a shorter survival rate.

**Table 3:** Clinicopathologic characteristics of NSCLC patients.

Characteristic	Cohort [n]
Total cases	30
Age, yr (median, range)	64(33‐75)
Gender	
Male	21
Female	9
Responsiveness	
Yes	10(33.3%)
No	20(66.7%)
Smoking history	
Yes	10(33.3%)
No	20(66.7%)
Histologic type	
ADC(Adenocarcinoma)	14(46.7%)
SCC(Squamous Cell Carcinoma)	12(53.3%)
TNM stage	
I	3(10%)
II	4(13.3%)
III	22(73.3%)
IV	1(3.3%)

### 2.6 CypA Attenuates NSCLC Chemosensitivity

**Figure 6:**
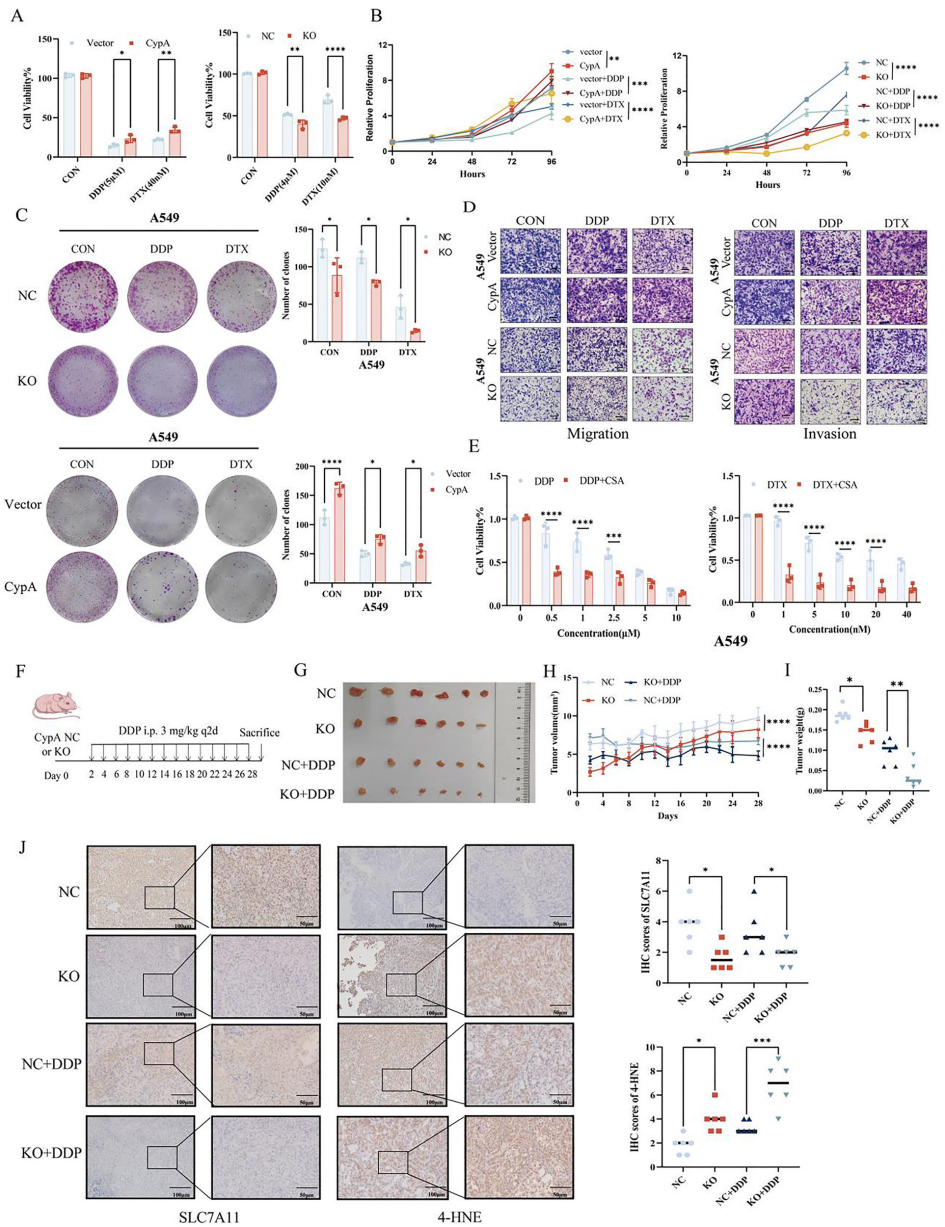
CypA attenuates NSCLC chemosensitivity. A,B) CCK‐8 assay analysis cell viability in A549 cells subjected to CypA knockout or overexpression and subsequently treated with DDP or DTX. C) Colony formation analysis cell viability in A549 cells subjected to CypA knockout or overexpression treated with DDP or DTX. D) Transwell analysis cell migration and invasion in A549 cells subjected to CypA knockout or overexpression and subsequently treated with DDP or DTX. E) CCK‐8 assay analysis cell viability in A549 cells treated with DDP or DTX combined with different concentrations of CSA. F‐J) NC or CypA knockdown A549 cells treated with DDP were implanted subcutaneously in nude mice (N = 6). (F) Xenograft model of subcutaneous tumor. Representative images of xenograft tumors are shown in (G). Weight change of nude mice is shown in (H), and the xenograft weight (I) are calculated. IHC images of SLC7A11 and 4‐HNE of xenograft tumors are shown in (J). Scale bar: 100 µm (left), 50 µm (right). Data are represented as the mean ± SD (*n* = 3). Statistical analysis was performed using Student’ s *t*‐test, *p < 0.05; **p < 0.01; ***p < 0.001; ****p < 0.0001.

To further validate the effect of CypA in NSCLC chemoresistance, cell viability and cell growth were analyzed in CypA‐manipulated parental or chemoresistant NSCLC cells. Loss of CypA sensitized NSCLC cells to DDP and DTX. Conversely, overexpression of CypA inhibited the sensitivity of NSCLC cells to these chemotherapeutic agents (**Figure** **6**A,B; Figure S3A‐B, Supporting Information). Additionally, under chemotherapeutic exposure, CypA overexpression enhanced cell migration and invasion in NSCLC cells. In contrast, CypA knockout significantly reduced the antitumor efficacy of DDP and DTX in A549 and H1299 cells (Figure 6C,D; Figure S3C,D, Supporting Information). Epithelial‐mesenchymal transition (EMT) is a cellular reprogramming process where epithelial cells acquire mesenchymal phenotypes, enhancing their migratory and invasive abilities, and it drives malignant cancer progression by promoting tumor growth, metastasis, and therapy resistance.^[^ ^32^ ^]^ overexpression of CypA enhanced EMT and CypA knockout is opposite in A549 and H1299 cells (Figure S3E, Supporting Information). Moreover, NSCLC cells were treated with CsA, the CypA inhibitor, in combination with chemotherapeutics enhanced the efficacy of DDP and DTX in NSCLC cells (Figure 6E; Figure S4A‐C, Supporting Information).

To further investigate the role of CypA in NSCLC chemoresistance in vivo, a NSCLC xenograft mouse model was generated by subcutaneously injecting control or CypA‐knockout A549 and H1299 cells (Figure 6F) into BALB/c nude mice. When treated with DDP, an obvious reduction in the tumor growth (Figure 6G–I; Figure S4D,E, Supporting Information) of the xenografts was observed in the CypA‐knockout group. Furthermore, the combination of CypA knockout and DDP treatment resulted in ferroptosis, as evidenced by weaker IHC staining of SLC7A11 and 4‐HNE (Figure 6J). Collectively, these results indicate that CypA facilitates NSCLC.

### 2.7 Targeting CypA Suppresses NSCLC Chemoresistance

**Figure 7:**
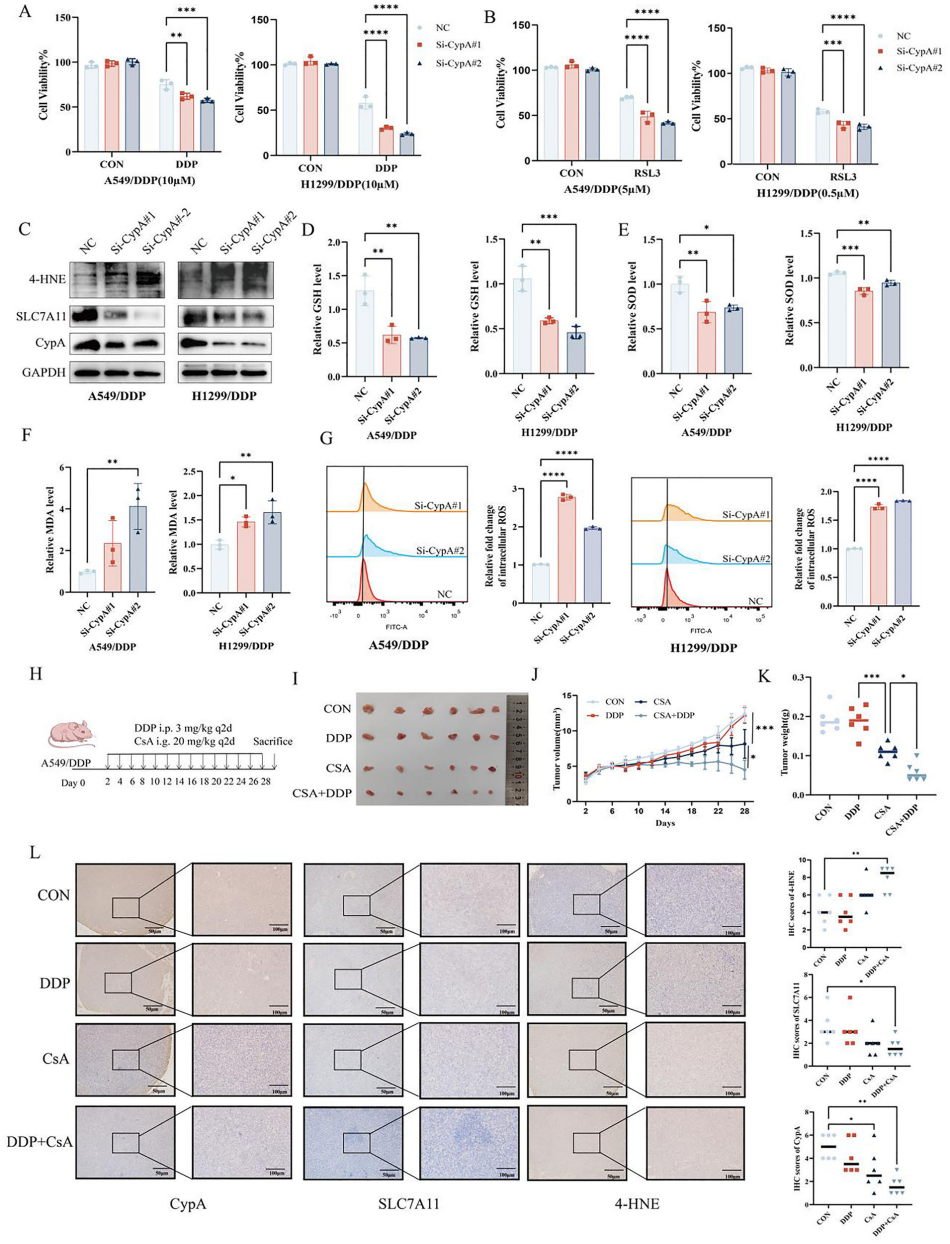
Targeting CypA suppresses NSCLC chemoresistance. A) CCK‐8 assay analysis cell viability in cisplatin‐resistant A549 and H1299 cell line (A549/DDP, H1299/DDP) treated with DDP after CypA knockdown. B) CCK‐8 assay analysis cell viability in A549/DDP and H1299/DDP cell line treated with RSL3 after CypA knockdown. C) Western blot analysis of 4‐HNEexpression in A549/DDP and H1299/DDP cell line after CypA knockdown. D) Expression of GSH in A549/DDP and H1299/DDP cell line after CypA knockdown. E) Expression of SOD in A549/DDP and H1299/DDP cell line after CypA knockdown. F) Expression of MDA in A549/DDP and H1299/DDP cell line after CypA knockdown. G) Flow cytometry analysis of ROS levels in A549/DDP and H1299/DDP cell line after CypA knockdown. H‐L) parental and resistant DDP A549 cells treated with DDP or/and CSA were implanted subcutaneously in nude mice (*N* = 6). (H) Xenograft model of subcutaneous tumor. Representative images of xenograft tumors are shown in (I). Weight change of nude mice is shown in (J), and the xenograft weight (K) are calculated. IHC images of CypA, SLC7A11 and 4‐HNE of xenograft tumors are shown in (L). Scale bar: 100 µm (left), 50 µm (right). Data are represented as the mean ± SD (*n* = 3). Statistical analysis was performed using Student’ s *t*‐test, *p < 0.05; **p < 0.01; ***p < 0.001; ****p < 0.0001.

Our previous studies have demonstrated that CypA is upregulated in chemotherapy resistance NSCLC and correlates with poor patient prognosis. To further elucidate the role of CypA in drug resistance, we performed CypA knockdown in resistant NSCLC cell lines (A459/DDP, A549/DTX, H1299/DDP, H1299/DTX). CCK8 assays showed that CypA knockdown significantly suppressed the proliferation of drug‐resistant cells (**Figure** **7**A; Figure S6A, Supporting Information). Moreover, transwell assays revealed that CypA depletion inhibited cell migration and invasion while reversing chemoresistance mediated EMT (Figure S5A,B, Supporting Information).

To confirm whether CypA facilitates NSCLC chemoresistance through ferroptosis, CCK8 assays showed that CypA knockdown significantly suppressed the proliferation of drug‐resistant cells treated with RSL3 (Figure 7B; Figure S6B, Supporting Information). Moreover, the levels of 4‐HNE, GSH, SOD, MDA, and ROS were measured in resistant NSCLC cell lines. As expected, CypA knockdown increased the levels of 4‐HNE, MDA, and ROS and decreased the levels of GSH and SOD in resistant NSCLC cell lines (Figure 7C–G; Figure S6C‐G, Supporting Information). To further confirm the clinical benefit to reverse chemoresistance effect of CsA and DDP in vivo, BALB/c nude mice were subcutaneously injected with A549/DDP cells and administered with DDP alone or in combination with CsA (Figure 7H). Analysis of the tumor size, tumor growth rate, and tumor weight revealed that CsA synergized with DDP in NSCLC treatment (Figure 7I–K). In line with this, IHC staining showed that the combined administration of CsA and DDP led to increased ferroptosis (Figure 7L). Collectively, these results suggest that CypA inhibitors synergistically enhance the antitumor effect in NSCLC with chemotherapy resistance.

## 3 Discussion

While early‐stage NSCLC is generally treated via surgery, the appreciable number of patients is diagnosed at advanced stages leading to a poor prognosis.^[^ ^33^ ^]^ Chemotherapy, particularly with cisplatin and taxanes (docetaxel or paclitaxel), is a main treatment for advanced cancers, but resistance to cisplatin and taxanes is becoming a major therapeutic challenge.^[^ ^2^ ^]^ In the present study, we identified CypA as a key player in chemotherapy resistance. CypA is significantly overexpressed in DDP/DTX‐resistant NSCLC and is closely linked to poor prognosis. Further investigations revealed that CypA contributes to DDP/DTX resistance by blocking ferroptosis via inhibition of SLC7A11. CypA stabilizes the expression of SLC7A11 at its K37 site by competitively binding to the E3 ligase TRIM3 of SLC7A11. The in vitro and in vivo models showed that genetic and pharmacological blockade of CypA restored the anti‐tumor effect of DDP. Therefore, our findings uncover an intimate relationship between CypA and DDP/DTX resistance, which provides a preclinical rationale for targeting CypA as a therapeutic strategy for patients with DDP/DTX resistant NSCLC.

CypA exhibits peptidylprolyl cis‐trans isomerase activity, which modulates substrate protein folding/stability and cell signaling. CypA has been identified as a dysfunctional protein in a variety of human cancers. CypA promotes the colonization and proliferation of multiple myeloma cells via binding to CD147.^[^ ^17^ ^]^ Furthermore, they identified CypA as an attractive target for the treatment of resistant multiple myeloma.^[^ ^34^ ^]^ Charalampos G have reported that CypA binds to and enhances activation of CrkII, which stimulate breast cancer cell migration.^[^ ^35^ ^]^ Our previous research has demonstrated elevated levels of CypA in ovarian cancer. Moreover, CypA drives EMT and ovarian tumor growth and metastasis via a TAF15/STAT5A/miR‐514a‐3p pathway.^[^ ^30^ ^]^ Although recent studies have established that CypA stabilizes NRF2 to drive lung cancer progression, the mechanisms linking CypA to ferroptosis regulation in chemotherapy‐resistant NSCLC remain unclear.^[^ ^29^ ^]^ In this study, we identified significant upregulation of CypA in NSCLC tissues and confirmed its tumor‐promoting role. Functional analyses revealed that CypA knockout markedly suppressed cancer cell proliferation, tumorigenesis, and EMT, while enhancing chemotherapy sensitivity. Notably, CypA depletion also elevated intracellular free iron, lipid peroxidation, and ROS accumulation, suggesting its pivotal role in suppressing this iron‐dependent cell death pathway. Chemotherapy resistance is frequently associated with heightened antioxidant defenses in cancer cells.^[^ ^36^ ^]^ Previous reports indicate that tumors may evade oxidative stress by inhibiting ferroptosis, thereby promoting therapy resistance.^[^ ^37^, ^38^ ^]^ Intriguingly, we observed elevated CypA levels in DDP/DTX‐resistant NSCLC cells. Crucially, CypA knockout reversed chemotherapy resistance by restoring ferroptosis sensitivity. The involvement of CypA in ferroptosis is supported by our proteomic results, which indicate that CypA regulates the expression of SLC7A11, and we confirmed by Westen blot that SLC7A11 was significantly decreased after CypA knockout, while no significant change was observed in GPX4. The cystine/glutamate antiporter SLC7A11 (also commonly known as xCT) functions to import cystine for glutathione biosynthesis and antioxidant defense and is overexpressed in multiple human cancers.^[^ ^39^ ^]^ Studies revealed that SLC7A11 overexpression in cancer cells promotes GSH biosynthesis and ferroptosis resistance maintaining cell survival under oxidative stress conditions.^[^ ^40^ ^]^ Of note, tumor tissue microarray and bioinformatics analysis indicated that CypA and SLC7A11 are vastly upregulated in patients with NSCLC. Herein, we demonstrate that CypA drives chemotherapy resistance in NSCLC by upregulating SLC7A11 to suppress ferroptosis.

Furthermore, the mechanism of CypA regulate SLC7A11 was explored. We found that CypA binds to SLC7A11 and stabilizes its expression. Accumulated evidences have demonstrated that the expression of SLC7A11 is regulated at multiple levels, including transcription, epigenetic regulation, and post‐translational modification. And we found that CypA not affect the SLC7A11 mRNA expression in NSCLC. Furthermore, we showed that CypA regulates SLC7A11 expression via the ubiquitination pathway. However, CypA is a peptidylprolyl cis‐trans isomerase activity protein by modulates substrate protein folding/stability and cell signaling. It has been reported that CypA stabilizes NRF2 by competitively binding to it against its E3 ubiquitin ligase KEAP1, thereby inhibiting NRF2 ubiquitination and degradation.^[^ ^29^ ^]^ Recent studies have identified several E3 ubiquitin ligases including SOCS2, HECTD3, ZRANB1, TRIM3, and CRL3KCTD10 that precisely regulate SLC7A11 stability through distinct mechanisms. In radiation‐treated hepatocellular carcinoma (HCC) cells, SOCS2 interacts with Elongin B/C to mediate K48‐linked polyubiquitination of SLC7A11, thereby enhancing radiosensitivity.^[^ ^19^ ^]^ The colon cancer field reveals that HECTD3 promotes SLC7A11 degradation to facilitate ferroptosis, whereas ZRANB1 maintains ferroptosis resistance.^[^ ^41^ ^]^ In non‐small cell lung cancer, TRIM3 specifically triggers K11‐linked polyubiquitination at lysine 37 (K37) of SLC7A11, inducing its proteasomal degradation.^[^ ^21^ ^]^ CRL3KCTD10‐mediated K48‐linked polyubiquitination of SLC7A11 is dynamically regulated by USP18 under cystine deprivation.^[^ ^42^ ^]^ Notably, our research reveals that CypA binds to SLC7A11 precisely at the K37 site on its N‐terminus. Given that TRIM3 also targets the K37 residue of SLC7A11 for interaction, we hypothesized that CypA and TRIM3 engage in competitive binding to SLC7A11. To test this, we conducted a series of co‐immunoprecipitation assays and functional experiments. The results consistently demonstrated that CypA effectively outcompetes TRIM3 for binding to SLC7A11, disrupting the TRIM3 and SLC7A11 interaction and thereby altering SLC7A11 ubiquitination and stability.

The FDA‐approved macrocyclic peptide CsA has been demonstrated to reverse drug resistance through its modulation of a broad spectrum of multidrug resistance proteins.^[^ ^43^ ^]^ In this study, we discovered an additional mechanism: CsA can enhance the antitumor efficacy of chemotherapeutic agents by overcoming CypA‐mediated resistance to ferroptosis in NSCLC. Our data demonstrate that CsA‐mediated modulation of CypA significantly increases NSCLC sensitivity to chemotherapy, thereby expanding our understanding of how CsA reverses drug resistance. Notably, throughout our study, none of the treated mice showed signs of severe weight loss, infection, or wound complications, even when multiple drugs were administered. This suggests that the combination of cisplatin (DDP) and a CypA inhibitor is well‐tolerated in vivo, indicating promising potential for clinical use in patients resistant to DDP or docetaxel (DTX). Several ongoing clinical trials further support the therapeutic potential of CsA in cancer treatment. For example, a Phase III trial (NCT00389870) is comparing the efficacy and toxicity of irinotecan alone versus irinotecan combined with CsA in patients with fluorouracil‐resistant advanced colorectal cancer (CRC). Although CsA is known for its immunosuppressive properties, previous clinical trials using various CsA administration protocols have consistently shown that its anticancer benefits outweigh the associated immunosuppression.^[^ ^44^ ^]^ The intermittent dosing or nano‐drug delivery represents an excellent strategy to mitigate these immunosuppressive side effects.^[^ ^45^, ^46^ ^]^ Specifically, administration of low‐dose CsA (e.g., 3‐day on/4‐day off cycles) may preserve its ability to modulate the CypA/SLC7A11 axis and induce ferroptosis in tumor cells, while reducing the sustained suppression of both systemic and intratumoral anti‐tumor immunity. At the same time nano‐delivery systems, tumor‐targeted nano‐carriers such as lipid nanoparticles or polymer‐based nanoparticles modified with tumor‐specific ligands can enrich CsA in tumor tissues. This approach minimizes the distribution of CsA in immune organs, thereby limiting its off‐target immunosuppressive effects while enhancing its local efficacy in regulating ferroptosis. The therapeutic potential of intermittent CSA treatment or nanoparticle‐based CypA delivery strategies for reversing NSCLC drug resistance needs to be further verified in subsequent in vivo and clinical experiments. Overall, our data provide a solid theoretical basis for the use of CSA to treat patients with DDP/DTX resistance in NSCLC. Therefore, the administration strategy based on CsA may need to be further optimized before it enters the clinic.

## 4 Conclusion

In summary, our study establishes CypA as a pivotal mediator of cisplatin/docetaxel (DDP/DTX) resistance in NSCLC through a novel ferroptosis suppressive mechanism. We demonstrate that CypA is overexpressed in chemotherapy‐resistant NSCLC and drives therapeutic evasion by competitively binding to the K37 site of SLC7A11, thereby blocking TRIM3‐mediated K11‐linked ubiquitination and proteasomal degradation of this critical ferroptosis regulator. These findings establish CypA as a therapeutic target, providing a preclinical basis for overcoming DDP/DTX resistance via ferroptosis reactivation in NSCLC.

## 5 Experimental Section

### Cell Culture

NSCLC cell lines A549(RRID:CVCL_0023), H1299(RRID:CVCL_0603), H460(RRID:CVCL_0459), human embryonic kidney HEK293T(RRID:CVCL_0063) and normal lung epithelial cells BEAS‐2B (RRID:CVCL_0168) were all purchased in 2023 from the Cell Bank of the Chinese Academy of Sciences (Shanghai, China) and cultured in 1640 or DMEM supplemented(Kaiji, Jiangsu, China) with 10% fetal bovine serum (Gibco, USA).All cell lines were free from mycoplasma contamination and kept in a humidified atmosphere containing 5% CO2 at 37 °C.

### Plasmids and siRNA

The SLC7A11, CypA, Ubiquitination and mutant plasmids were obtained from GENG (Shanghai, China). Cells were transfected with the indicated plasmids and siRNAs with Lipofectamine 3000 (Invitrogen) according to the manufacturer's instructions.

### Generation of CypA Knockout and Overexpression NSCLC Cell Lines

In this study, CypA was stably overexpressed or Knockout in NSCLC cell lines. The full‐length cDNA of CypA was cloned into lentivirus vectors at certain sites and CypA Knockout cell lines were constructed by CRISPR‐Cas9 (Corues, Jiangsu, China). When the cell fusion density reached ≈30% confluence, it infected the NSCLC cells with CypA Knockout lentiviral vector, Lentivirus (Cas9‐Puro)/U6‐PPIA‐KO3 or overexpressed lentiviral vectors, Lentivirus (GFP‐Puro)/CMV‐PPIA. One week after transfection, positive cells were screened with puromycin (Beyotime, Shanghai, China) and verified by Western blot.

### Cell Counting Kit‐8 (CCK‐8)

NSCLC cells (1.5 × 10^3^) were seeded in 96‐well plates with 5 replicates, drugs were added after cell adhesion, and incubated for 72 h, then 10 µL CCK‐8 solution (vicmed, Jiangsu, China) was added to each well under light‐proof conditions. The number of cells in the proliferation experiment plate was 1.0× 10^3^ per well, and the absorbance was measured at 24, 48, 72, and 96 h after plate laying. Cell viability was calculated by normalizing the absorbance at 450 nm of the experimental groups to that of the negative control group.

### Colony Formation

Approximately 1000 cells were seeded in 6‐well plates, and incubated at 37 °C for 10 days, washed three times with phosphate‐buffered saline (PBS), fixed by 4% paraformaldehyde for 10 min, and then stained with 0.1% crystal violet for 10 min. Then, the 6‐well plates were gently washed with water and air‐dried at room temperature. Photographs were taken under a microscope and the units of colony formation were counted using ImageJ software (1.52v).

### Transwell Migration and Invasion Assay

The difference between the trans‐well migration and invasion assay was that the invasion assay requires pre‐coating of the matrigel (Corning Costar, New York, USA) diluted with serum‐free medium in the upper layer of the chamber, and in addition. A total of 4 × 10^3^ NSCLC cells suspended in serum free 1640 dilution were seeded into the upper chamber, and the lower chamber contained 10% FBS serves as a chemoattractant. After 24 or 48 h of incubation at 37 °C, the chamber was fixed with 4% paraformaldehyde and stained with a 0.1% crystal violet. After removing the cells on the upper surface of the insert with cotton swabs, five random visual fields were selected for quantification under the microscope.

### Western Blot Analysis

Total protein was extracted with cell RIPA lysis buffer (Kaiji, Jiangsu, China) supplemented with the proteinase inhibitors (Vicmed, Xuzhou, China) and phosphatase inhibitor (Proteintech, Chicago, USA). Cells were lysed in cell lysis buffer and protein concentrations in the lysates were determined with BCA protein determination kit (Kaiji, Jiangsu, China). Protein samples were separated by sodium dodecyl sulfate‐polyacrylamide gel electrophoresis (SDS‐PAGE) and transferred onto polyvinylidene difluoride (PVDF) membranes (Millipore, Bedford, MA, USA). Membranes were incubated with primary antibodies followed by secondary antibodies, and visualized by using an ECL kit (Yeasen, Shanghai, China) and scanned by Bio‐Rad (Hercules, CA, USA).The primary antibodies used for western blot analysis were listed as follows: E‐cadherin (Proteintech, 20874‐1‐AP), N‐cadherin (Proteintech, 22018‐1‐AP), Vimentin (Affinity, BF8006), Rabbit anti‐CypA (Affinity, DF6175), mouse anti‐CypA(Santa cruze, SC‐134310), SLC7A11 (Abcam, AB307601), 4‐HNE (Bioss, D50444), Flag (Proteintech, 20543‐1‐AP), HA (Proteintech, 66006‐2‐Ig), Ub (Proteintech, 10201‐2‐AP), TRIM3 (Proteintech, 28392‐1‐AP), GAPDH (Proteintech, 60004‐1‐Ig).

### Protein Stability Assay

To measure the half‐life of SLC7A11, cells were treated with 100 µM protein synthesis inhibitor cycloheximide (CHX) (MedChemExpress, New Jersey, USA) for indicated times. Western blot was performed to measure protein levels.

### Structural Modeling and Docking Analysis

The human CypA structure was obtained from UniProt (PDB ID: 1AWQ), while the SCL7A11 was modeled using AlphaFold3, with the highest‐confidence model (pLDDT > 85) selected for analysis. Both structures were prepared in UCSF Chimera v1.17.3: CYPA was cleaned by removing water and heteroatoms, with hydrogen atoms added at pH 7.4, while the SCL7A11 fragment underwent energy minimization using the MMFF94 force field and Gasteiger charge assignment. Molecular docking was performed using Dock6 v6.11. Structural interactions were visualized in PyMOL, with CypA shown as a gray cartoon, the SCL7A11 fragment as slate sticks, and interacting residues in salmon. This workflow was purely computational; no experimental validation (e.g., crystallization) was performed, and the model serves as a hypothesis for mechanistic interpretation.

### Co‐Immunoprecipitation

Cells were harvested and lysed on ice in a lysis buffer. Cell lysates were incubated with primary antibodies at 4 °C overnight. After incubation, the Magnetic beads (MedChemExpress, New Jersey, USA) were added to the EP tube and stirred for 2 h at 4 °C. Then, the beads were washed four times with pre‐cooled PBS, and the precipitates were eluted with a 5× loading buffer. After boiling at 100 °C for 5 min, proteins were detected by SDS‐PAGE and Western blot.

### Surface Plasmon Resonance (SPR)

The SPR technique, a method for analyzing real‐time biomolecular interactions without labeling, was used to evaluate the binding affinity between recombinant human CypA and SLC7A11 proteins. The SLC7A11 protein was immobilized directly on a CM5 chip (BR‐1005‐30, China). After incubation, a concentration gradient of CypA was injected over the SLC7A11‐coated CM5 sensor chip. To investigate whether CypA influences the binding between SLC7A11 and TRIM3, both SLC7A11 and TRIM3 proteins were introduced along with a gradient of CypA into the flow system. Binding interactions were analyzed using BIAcore T200 evaluation software (v.2.0, GE Healthcare, USA). The experimental procedure followed methodologies described in previous studies.^[^ ^47^ ^]^

### In Vitro Ubiquitination Assay

HEK293T cells were transfected with empty vector or Flag‐SLC7A11 and Ub plasmids. Forty‐eight hours after transfection, and add 10 µL of MG132 (MedChemExpress, New Jersey, USA) act for 24 h, The Flag antibody was pulled down for coimmunoprecipitation, following the same steps as coimmunoprecipitation.

### Mass Spectrometry (MS)

Collect the cells of CypA KO and the NC cells, the collected cell lysate was swirled and ultrasonically broken. After standing at 4 °C for 30 min, the supernatant was centrifuged at 14000 × g and the protein concentration was measured. Then 0.1% formic acid solution was added to redissolve and LC–MS/MS analysis was performed. The acquired MS raw files were analyzed by MaxQuant environment (version 1.6.5). The tandem MS was searched against the UniProt proteome. The mass difference of precursor and fragment ions was set to 10 ppm and 0.5 Da, respectively. The false detection rates for peptides and proteins were all set below 0.01. All the other parameters in MaxQuant were set to default values.

### Real‐Time Quantitative PCR (RT‐qPCR)

Total RNA was extracted by using TRIzol reagent (Tiangen, Beijing, China). The PrimeScript RT Reagent Kit (Servicebio, Hubei, China) was used to perform reverse transcription. The real‐time PCR reactions were performed in triplicate on a Light Cycle 96 Real‐Time PCR Detection System (Roche, Basel, Switzerland), and gene expression was analyzed according to the 2−ΔΔCq relative quantification method.

### ROS Assay

The ROS assay Kit (S0033, Beyotime) was used to determine the levels of ROS in tumor cells. The cells were plated in six‐ well plates at a density of 2 × 10^5^ cells/well. After transfecting the cells with SiRNA, they were incubated with 10 µM DCFH‐DA (for ROS determination), the fluorescence intensity of ROS was tested by flow cytometry (FACSCalibur, BD Biosciences).

### MDA Assay

The MDAassay Kit (Jiancheng Jiangsu, China) was used to determine the levels of ROS in tumor cells. In brief, ≈2 × 10^6^ cells to be tested were collected. After treatment with MDA‐specific lysis buffer and centrifugation, 200 µL of the supernatant was added to the chromogenic solution and incubated at 95 °C for 90 min. After the temperature drops to room temperature, measure the absorbance to calculate the MDA content.

### SOD Assay

The SOD assay Kit (Jiancheng Jiangsu, China) was used to determine the levels of ROS in tumor cells. The cells to be tested were treated with cell lysis buffer, and the supernatant was centrifuge and added to the enzyme working solution and substrate reaction solution. The cells were incubated for 20 min and the SOD concentration was calculated by absorbance.

### GSH Assay

The Glutathione Assay Kit (Jiancheng Jiangsu, China) was used for the detection of total GSH according to the manufacturer's instructions. Collect the cells to be tested in a centrifuge tube, add the proteinizing reagent, let it stand at room temperature for 5 min, and repeat the shaking. After centrifugation, collect the supernatant and add it to the chromogenic solution. Measure the absorbance and calculate the GSH concentration.

### Fe^2+^ Assay

The Fe^2+^ assay Kit (Meilunbio dalian, China) was used to determine the levels of Fe^2+^ in tumor cells. 1.5 × 10^4^ cells to be tested were spread in 48‐well plates, and added 10µL fluorescent probe the next day. Observe the fluorescence intensity under a fluorescence microscope.

### Immunofluorescence

Cells were fixed in 4% paraformaldehyde and permeabilized with 0.1% Triton X‐100. Subsequently, the cells were blocked with 5% BSA and incubated with primary antibodies overnight at 4 °C. Then, the cells were incubated with the corresponding secondary antibodies avoiding light for 1 h and DAPI was applied for 10 min. Finally, images were captured by confocal laser scanning microscopy (Leica STELLARIS 5, Weztlar, Germany). Image analysis was performed using ImageJ software.

### Human Tissues and Immunohisthemistry (IHC)

Human NSCLC tissues were obtained from the Affiliated Hospital of Xuzhou Medical University. This study was approved by the Medical Ethics Committee of the Affiliated Hospital of Xuzhou Medical University (XYFY2024‐KL031‐01). Informed consent for tissue analysis was obtained before the surgery. All the research was performed in accordance with government policies and the Helsinki declaration. IHC staining of human tissues, tissue chip, xenograft tumors were performed by Servicebio Technology (Hubei, China). Tissues were fixed in 10% formalin, embedded in paraffin, and then cut into slices with a thickness of 4 µm. Antigen retrieval was conducted before incubation with appropriate antibody at 4 °C overnight. The next day, the secondary antibody was incubated and re‐stained with the chromogenic solution and hematoxylin, and then observed under a microscope. Evaluation of IHC staining was conducted by two independent pathologists using the H‐score method as previously described.^[^ ^30^ ^]^

### Animal Study

Six‐week‐old male nude mice were purchased from the Central Laboratory of Animal Science of Xuzhou Medical University, and the animal facility was SPF at 20–24 °C. For subcutaneous tumor xenograft, 2 × 10^6^ A549 or A549/DDP cells were injected under the skin of nude mice. All mice were randomly divided into four groups. When the tumor volume reached 150‐200 mm^3^, the mice were treated with DDP alone (3 mg kg^−1^, every 2 days, ip) or combined with CSA (4 mg kg^−1^ every 2 days, oral gavage). Record the changes of the tumor. At the end of the experiment, all mice were sacrificed, the tumor weight was recorded, and the tissues were collected for IHC staining. This study was approved by the Institutional Animal Care and Use Committee of Xuzhou Medical University (202309T009).

### Statistical analysis

The data were shown as the mean ± SD of at least three independent experiments. Statistical analysis was performed using GraphPad software 9.0. Student's t‐test was used for comparisons between two groups, and one‐way analysis of variance was used for comparisons among more than two groups. The log‐rank test was used to determine the statistical significance of Kaplan‐Meier survival curves. A value of p < 0.05 was considered statistically significant (*p < 0.05, **p < 0.01, ***p < 0.001, and ****p < 0.0001). All in vitro experiments were performed at least three replicates and the data presented were from one representative experiment.

### Ethical Approval

All participants provided informed consent. All human tissue research in this study had the approval of ethics committees of the Affiliated Hospital of Xuzhou Medical University (Xuzhou, China). All of the animal experiments were performed by the relevant guidelines and regulations and were approved by the Institutional Animal Care and Use Committee (IACUC) of Xuzhou Medical University.

## Conflict of Interest

The authors declare no conflict of interest.

## Author Contributions

Z.W., A.L., Z.S., and X.L. contributed equally to this work. T.L., and H.Z. designed, supervised the study, and revision and final approval of the manuscript. Z.C.W. and T.L. contributed to data research, analysis, and manuscript writing. A.L., Z.W.S., and B.Y.Z. contributed to experiment implementation. X.M.L., Y.G., Z.Q.C. and Y.H.L. contributed to the sample collection and data analysis. All the authors have read the manuscript and provided useful comments.

## Supporting information



Supporting Information

Supporting Table

## Data Availability

The data that support the findings of this study are available from the corresponding author upon reasonable request.
