# Supplementary material for: CypA Mediates Non‐Small Cell Lung Cancer Chemoresistance by Attenuating Ferroptosis via Stabilizing SLC7A11
Source: Adv Sci (Weinh). 2025 Nov 7;13(4):e11947. doi: 10.1002/advs.202511947 (PMC12822425; doi:10.1002/advs.202511947)
Supplement: Supplementary file 1 — Supporting Information [file ADVS-13-e11947-s001.docx]

Supporting Information

CypA mediates non-small cell lung cancer chemoresistance by attenuating ferroptosis via stabilizing SLC7A11

***Authors:*** *Zhongcheng Wang^1,3^, An Li^1,2,5^, Ziwei Song^1,2^, Xiangming Liu^4^, Yong Ge^4^, Zhiqiao Chen^4^, Yuhui Liu^1,2^, Boyu zhang^4^, Hao Zhang*^4^, Ting Lan*^1,2^*

Figure S1


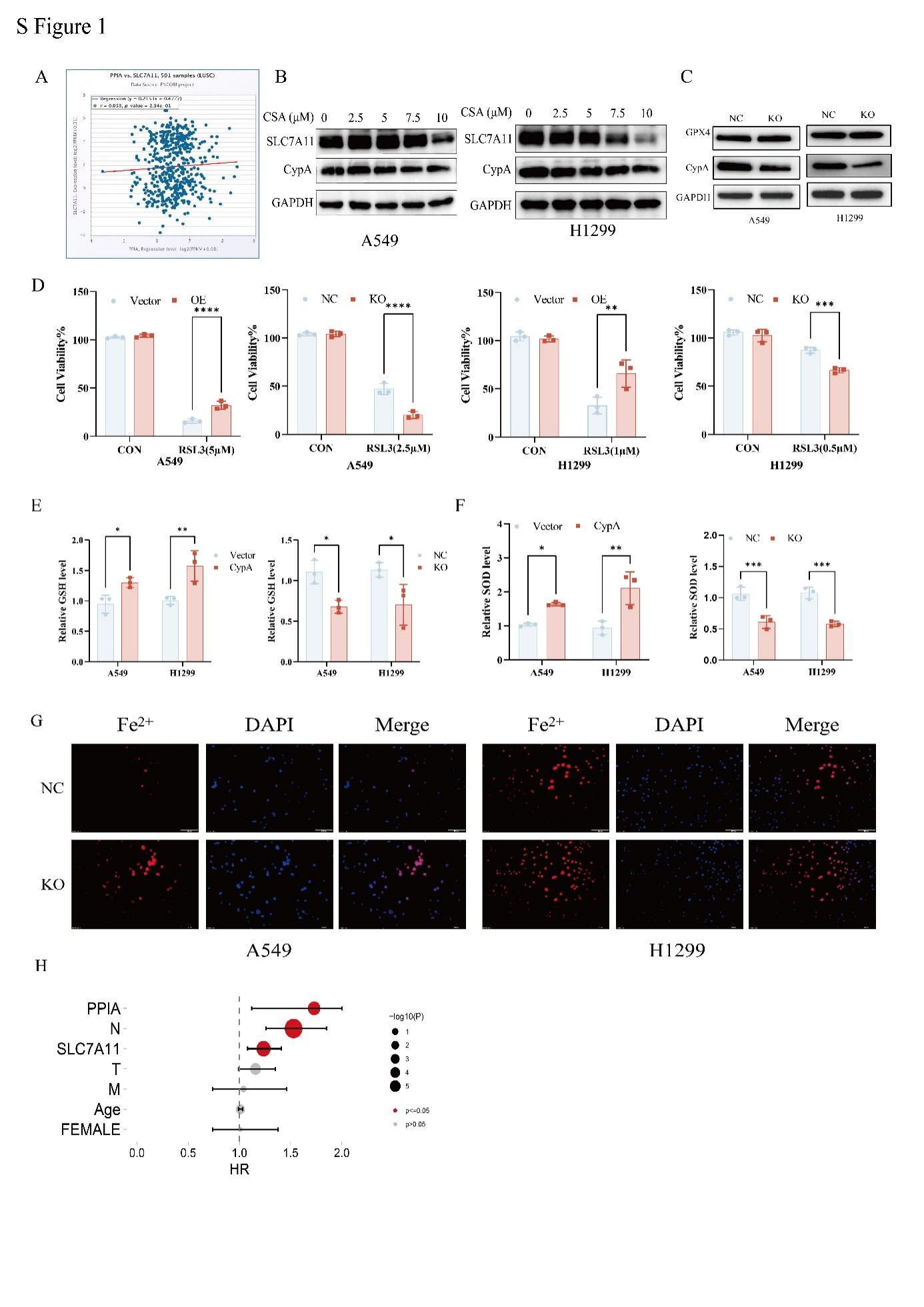


**A** ENCORI database predicts the correlation between SLC7A11 and CypA in NSCLC. **B** Western blot analysis the expression of SLC7A11 in NSCLC cells treated with different concentration CSA. **C** Western blot analysis GPX4 expression after CypA overexpression or knockout in A549 and H1299. **D** CCK-8 assay analysis cell viability in A549 and H1299 cells CypA knockout or overexpression treated with RSL(5µM). **E** Expression of GSH after CypA overexpression or knockout in A549 and H1299. **F** Expression of SOD after CypA overexpression or knockout in A549 and H1299. **G** Immunofluorescence analysis of Fe²⁺ levels after CypA knockout in A549 and H1299. **H** Figure X. Forest plot illustrating the hazard ratios (HRs) and 95% confidence intervals (CIs) from multivariable Cox regression analysis for the association between various clinicopathological factors and gene expression levels (PPIA, SLC7A11, with expression values normalized as log (TPM + 1), T stage, M stage, age, and gender, and survival outcomes in NSCLC. Data are represented as the mean ± SD (n = 3). Statistical analysis was performed using Student’ s t-test, **p* < 0.05; ***p* < 0.01; ****p* < 0.001; *****p* < 0.0001.

Figure S2


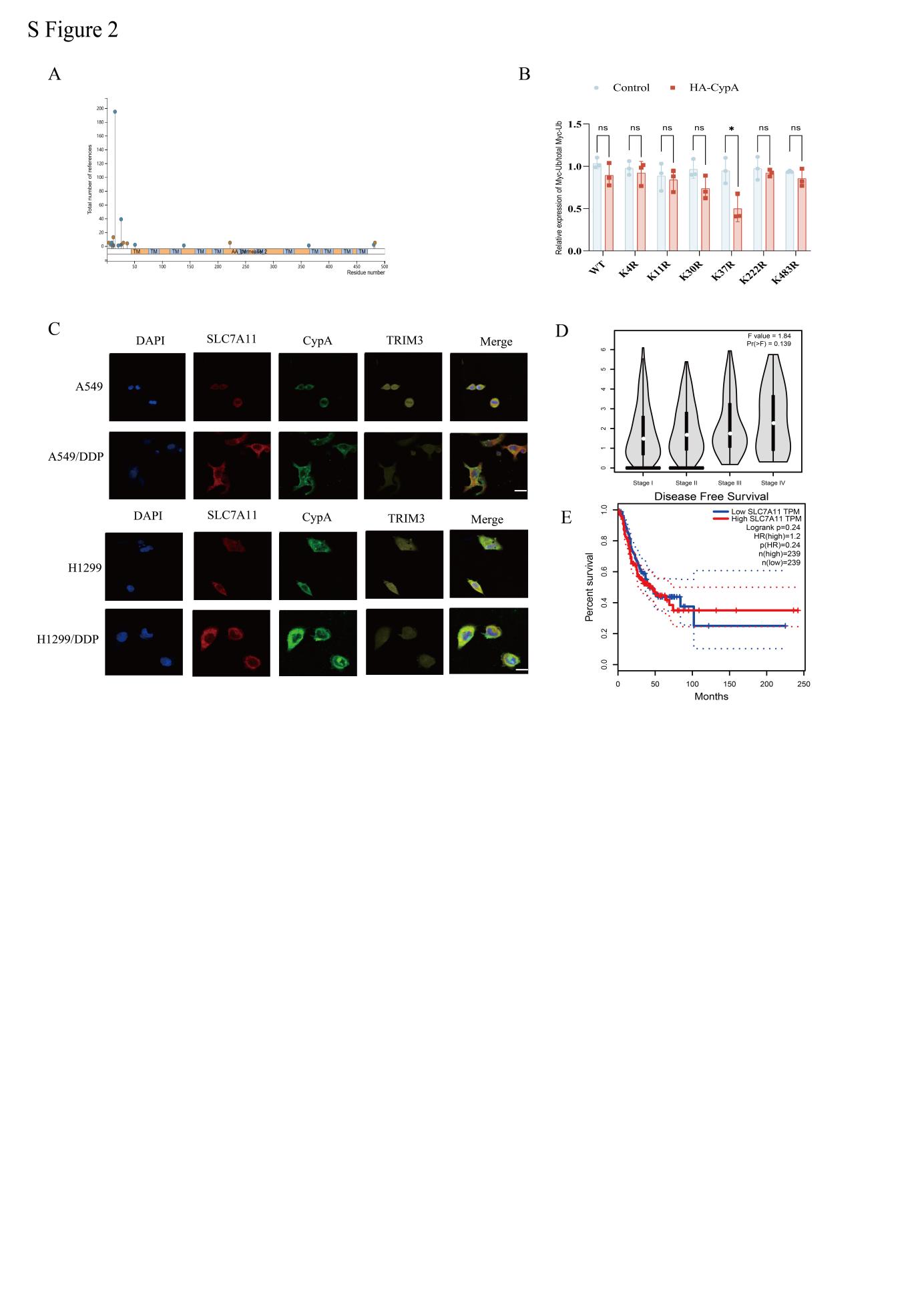


**A** PhosphositePlus v6.7.9 software predicts SLC7A11 ubiquitination sites. **B** Statistics of ubiquitination detected by western bolt after different mutants of SLC7A11.**C** Confocal assay showing co-localization of CypA (green), xCT (red) and TRIM3 (yellow) in A549 and H1299 cells. Nuclei were counterstained with DAPI (blue). Scale bar: 10 μm. **D** GEPIA database analysis SLC7A11 expression levels in NSCLC patients at different stage. **E** Kaplan-Meier analysis of the progression-free survival of NSCLC patients associated with SLC7A11 expression. Data are represented as the mean ± SD (n = 3). Statistical analysis was performed using Student’ s t-test, **p* < 0.05.

Figure S3


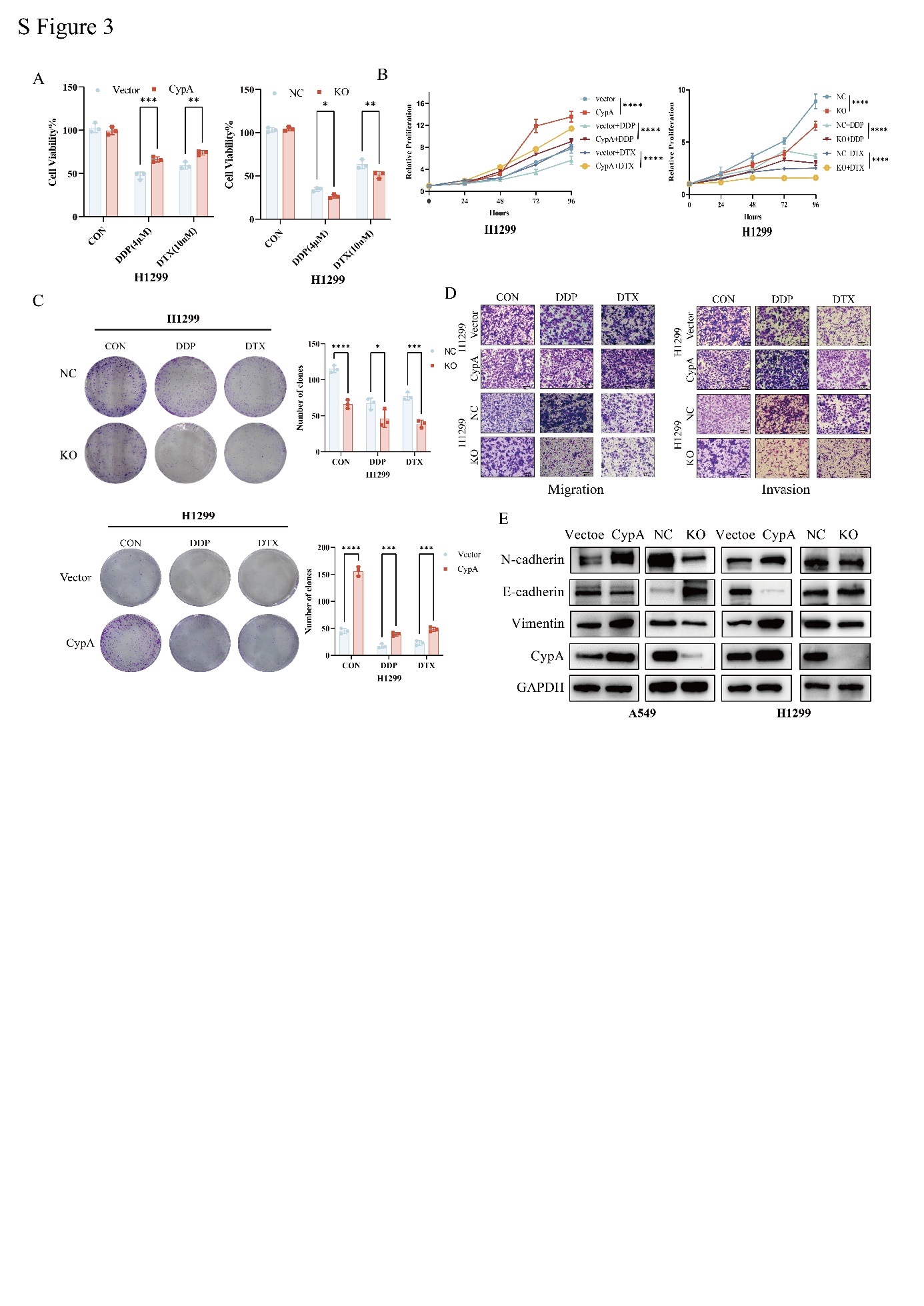


**A-B** CCK-8 assay analysis cell viability in H1299 cells subjected to CypA knockout or overexpression and subsequently treated with DDP or DTX. **C** Colony formation analysis cell viability in H1299 cells subjected to CypA knockout or overexpression treated with DDP or DTX. **D** Transwell analysis cell migration and invasion in H1299 cells subjected to CypA knockout or overexpression and subsequently treated with DDP or DTX. **E** Western blot anlysis N-cadherin, E-cadherin, and Vimentin expression after CypA overexpression or knockout in A549 and H1299. Data are represented as the mean ± SD (n = 3). Statistical analysis was performed using Student’ s t-test, **p* < 0.05; ***p* < 0.01; ****p* < 0.001; *****p* < 0.0001.

Figure S4


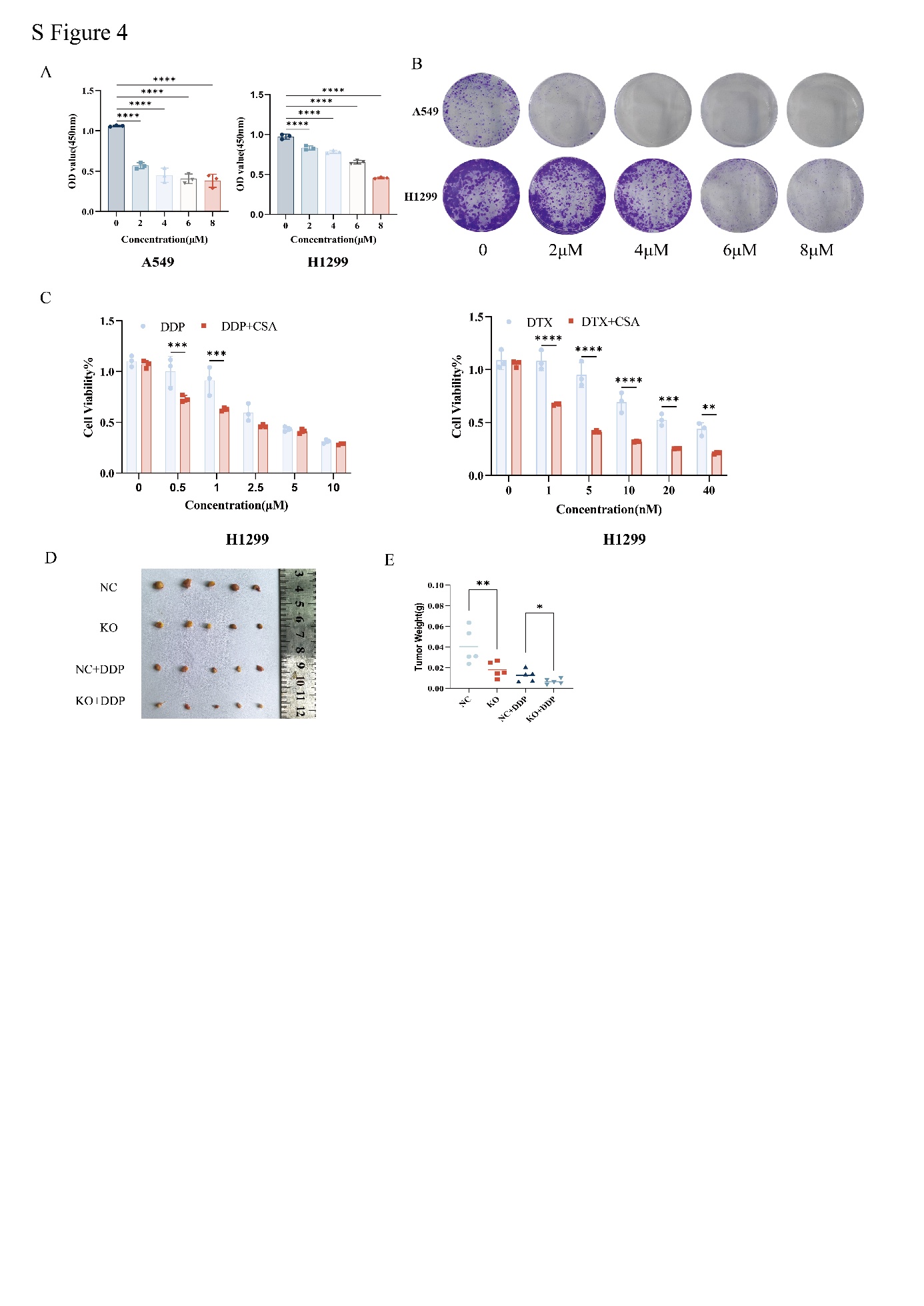


**A** CCK-8 assay analysis cell viability in A549 and H1299 cells treated with different concentration CSA. **B** Colony formation analysis cell viability in A549 and H1299 cells treated with different concentration CSA. **C** CCK-8 assay analysis cell viability in H1299 cells treated with CSA (4µM) combined with different concentrations of DDP or DTX. **D** NC or CypA knockdown H1299 cells treated with DDP were implanted subcutaneously in nude mice(N=5). **E** The xenograft weight are calculated. Data are represented as the mean ± SD (n = 3). Statistical analysis was performed using Student’ s t-test, ***p* < 0.01; ****p* < 0.001; *****p* < 0.0001.

Figure S5


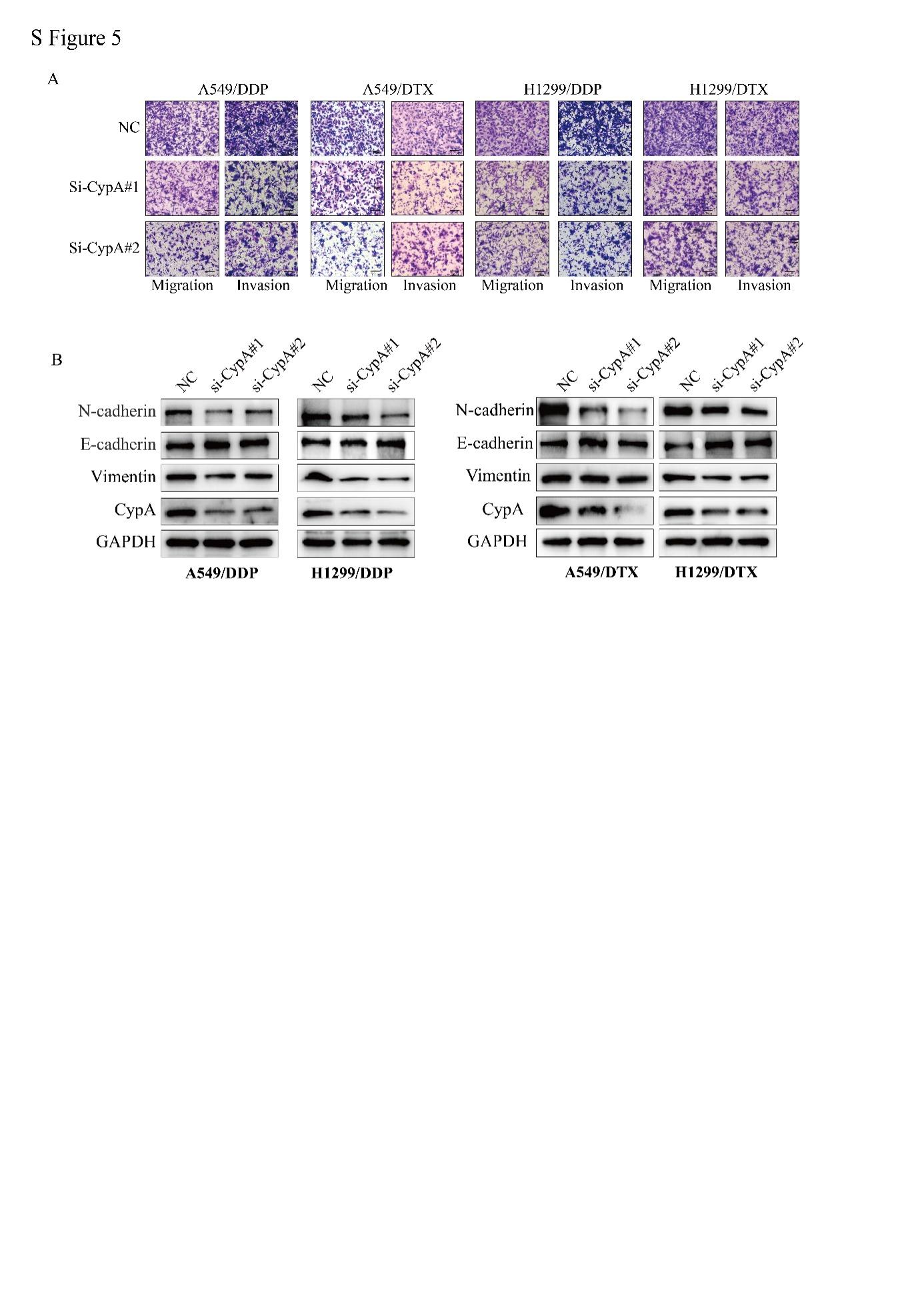


**A** Transwell analysis cell migration and invasion in cisplatin/paclitaxel-resistant A549 and H1299 cell line (A549/DDP, A549/DTX, H1299/DDP, H1299/DTX) treated with DDP/DTX after CypA knockdown. **B** Western blot anlysis N-cadherin, E-cadherin, and Vimentin expression in cisplatin/paclitaxel-resistant A549 and H1299 cell line (A549/DDP, A549/DTX, H1299/DDP, H1299/DTX) treated with DDP/DTX after CypA knockdown.

Figure S6


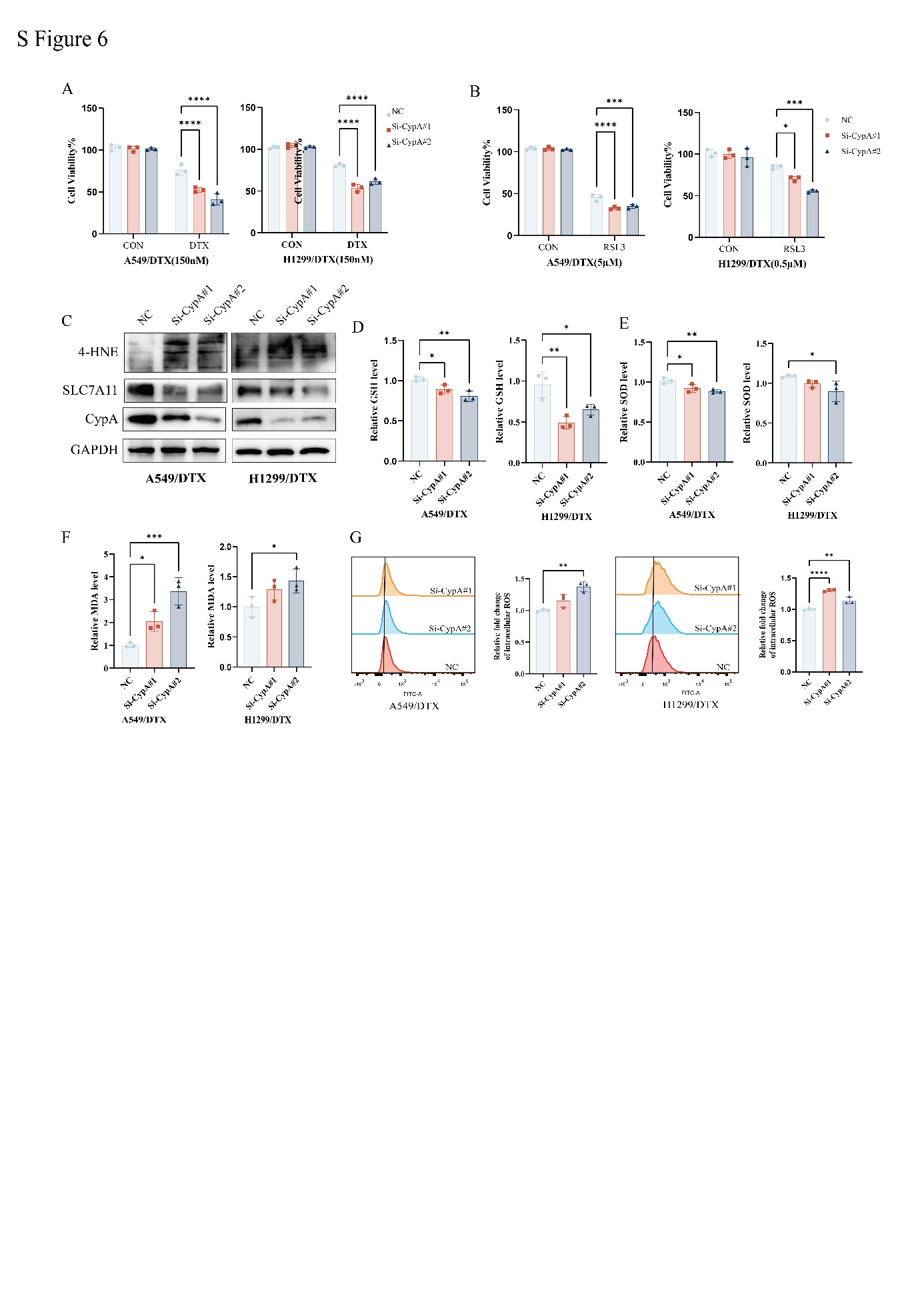


**A** CCK-8 assay analysis cell viability in paclitaxel-resistant A549 and H1299 cell line (A549/DTX, H1299/DTX) treated with DTX after CypA knockdown. **B** CCK-8 assay analysis cell viability in A549/DTX and H1299/DTX cell line treated with RSL3 after CypA knockdown. **C** Western blot analysis of 4-HNE expression in A549/DTX and H1299/DTX cell line after CypA knockdown. **D** Expression of GSH in A549/DTX and H1299/DTX cell line after CypA knockdown. **E** Expression of SOD in A549/DTX and H1299/DTX cell line after CypA knockdown. **F** Expression of MDA in A549/DTX and H1299/DTX cell line after CypA knockdown. **G** Flow cytometry analysis of ROS levels in A549/DTX and H1299/DTX cell line after CypA knockdown. Data are represented as the mean ± SD (n = 3). Statistical analysis was performed using Student’ s t-test, **p* < 0.05; ***p* < 0.01; ****p* < 0.001; *****p* < 0.0001.
